# The association of gestational age and birthweight with blood pressure, cardiac structure, and function in 4 years old: a prospective birth cohort study

**DOI:** 10.1186/s12916-023-02812-y

**Published:** 2023-03-20

**Authors:** Bowen Du, Hualin Wang, Yujian Wu, Zhuoyan Li, Yiwei Niu, Qianchuo Wang, Lin Zhang, Sun Chen, Yurong Wu, Jihong Huang, Kun Sun, Jian Wang

**Affiliations:** 1 Department of Pediatric Cardiology, Xinhua Hospital Affiliated To Shanghai Jiao Tong University School of Medicine, Yangpu District, No.1665, Kongjiang Road, Shanghai, 200092 China; 2 The International Peace Maternity and Child Health Hospital, School of Medicine, Shanghai Jiao Tong University, Shanghai, China; 3 Shanghai Key Laboratory of Embryo Original Diseases, Shanghai, China

**Keywords:** Gestational age, Birthweight, Blood pressure, Left ventricle

## Abstract

**Background:**

Current evidence relating birthweight and gestational age to cardiovascular risk is conflicting. Whether these factors have independent or interactive impacts on cardiovascular parameters during early childhood remains unclear. The goal of this study was to explore whether there were any independent and interactive effects of gestational age and birthweight on blood pressure, left ventricle (LV) structure, and function in 4 years old.

**Methods:**

This study included 1194 children in the Shanghai Birth Cohort from 2013 to 2016. Information about the mothers and children was recorded at time of birth using a questionnaire. Follow-up measurements, including anthropometric, blood pressure, and echocardiography, were taken between 2018 and 2021, when the children were 4 years old. Multiple linear or logistic regressions and restricted cubic spline were used to explore the association of birthweight and gestational age with cardiovascular measurements.

**Results:**

Gestational age had a significant negative correlation with both systolic blood pressure [*β* =  − 0.41, 95% CI: (− 0.76, − 0.07)] and mean arterial pressure [*β* =  − 0.36, 95%CI: (− 0.66, − 0.07)]. The risk of prehypertension decreased with increased gestational age [OR = 0.54, 95% CI: (0.32, 0.93)]. The relationship between birthweight with blood pressure was U-shape (*P* for non-linear < 0.001). The wall thickness, volume, mass, and cardiac output of LV increased with birthweight, though the ejection fraction [*β* =  − 1.02, 95% CI: (− 1.76, − 0.27)] and shorten fraction [*β* = 0.72, 95% CI: (− 1.31, − 0.14)] decreased with birthweight. The risk of LV hypertrophy was not associated with birthweight [OR = 1.59, 95% CI: (0.68, 3.73)].

**Conclusions:**

In this study, we found different associations of birthweight and gestational age with cardiovascular measurements in the offspring at 4 years old. Gestational age influenced blood pressure independent of birthweight. Heart size and function at 4 years old was influenced mostly by birthweight and not by gestational age.

**Supplementary Information:**

The online version contains supplementary material available at 10.1186/s12916-023-02812-y.

## Background

The risk of cardiovascular disease (CVD) in adulthood may be determined from early life [1] and is potentially influenced by both the intrauterine environment and early childhood development [2], which may be reflected in birthweight (BW) and gestational age (GA). The potential influence of BW and GA on the cardiovascular system is complicated and controversial. Recent studies have found that children with low BW or GA are linked to increase risk of CVD, such as hypertension [3, 4], heart failure [5], hypertrophy [6], ischemia disease [7, 8], or arrhythmias [9] in adolescence or in adulthood [10, 11]. Other studies have found that children with large BW have an increased future risk of CVD or hypertension [12–14]. Conversely, a British study suggested that large BW was associated with a lower future blood pressure (BP) [15]. Studies focused on preterm birth have found that it might increase the risk of hypertension or other CVD from childhood to adulthood [5, 7, 16, 17]. Any potential non-linear relationship between GA or BW and cardiovascular measurements remains unknown.

BW is strongly, but not only, influenced by GA. Children with low BW are usually preterm birth. Several studies have focused only on the simple linear correlation between BW and GA with cardiovascular indexes [16, 18, 19]. Other studies have focused primarily on participants per se, such as only prematurity or low BW, often without accounting for BW and GA independently [20]. Juonala et al. [21] observed increased BP levels only among preterm low BW participants and not in full term participants. These results suggested that GA might influence BP independently from BW. Other studies have focused on CVD risk in adolescence or adulthood [5, 7, 22], though the influence of BW and GA on the cardiovascular system may start in childhood. Therefore, studies investigating these risks during the early childhood period are still valuable. Additionally, evidence on the independent or interactive regulation of BW and GA on cardiovascular risk remains limited.

This study aimed to explore the independent and interactive associations of BW and GA with BP, left ventricle (LV) structure, and function in early childhood, based on detailed BP and echocardiography evaluation from a birth cohort in China.

## Methods

### Participants

The Shanghai Birth Cohort (SBC) is an ongoing prospective cohort study conducted in six collaborating hospitals in Shanghai, China. A detailed description of the cohort has been provided elsewhere [23]. In this study, 1194 mother–child pairs joined our cardiovascular cohort between 2013 and 2016. The maternal self-reported questionnaires were conducted after enrollment and during pregnancy with the help of trained staff. Information on maternal demographic and sociodemographic characteristics (e.g., maternal income, race, education level) and lifestyle factors (e.g., passive smoking or alcohol drinking status during pregnancy) were collected using structured questionnaires. The history of hypertensive disorders in pregnancy (HDP) and gestational diabetes mellitus (GDM) was collected using structured questionnaires or extraction of inpatient history from medical records. Follow-up measurements (including weight, height, blood pressure and echocardiography) of children were carried-out between 2018 and 2021 when they were 4 years old. Children with congenital heart disease or other birth defects and those lost to follow-up, who were uncooperative, or without all available records were excluded. A total of 943 children were finally included in all analyses. Ethical approval was granted by the Ethical Committee of Xinhua Hospital affiliated to Shanghai Jiao Tong University School of Medicine (No. XHEC-C-2013–001-2). All parents or guardians of participants signed written informed consent forms prior to enrollment.

### Anthropometric measurement

The BW and GA were recorded after birth and extracted from the inpatient medical history of the pregnant women or from pediatric medical records. Between 2018 and 2021, when the children were 4 years old, their gender, height, and weight were measured and recorded. BMI was calculated by weight (kg)/height (cm)^2^. Preterm was defined as newborns with GA < 37 weeks. Term was defined as children with GA between 37 and 42 weeks. The BW groups were classified as follows: low birth weight (LBW) group < 2500 g, normal birthweight (NBW) group 2500–4000 g, and macrosomia group with birthweight > 4000 g.

### Blood pressure (BP)

Systolic BP (SBP) and diastolic BP (DBP) of children were assessed by a single, trained staff member on the left arm at heart level and with the appropriate cuff size for arm circumference while they were in the supine position. The OMRAN HBP-1300 automatic BP device (Omron Healthcare, Guangzhou, China) was used. Three measurements were taken at 5 min intervals once each child had calmed down. The mean of the three measurements was used in all analyses. Mean arterial pressure (MAP) was calculated as MAP = DBP + (SBP-DBP)/3. The 90th and 95th percentile of SBP and DBP for the sex and height were defined according to the Chinese standard [24]. Prehypertension was defined as P95th > SBP and/or DBP ≥ P90th. Hypertension was defined as SBP and/or DBP ≥ P95th.

### Echocardiography

Transthoracic echocardiography examinations were performed on the children by trained operators according to the American Society of Echocardiography recommendations [25] and using the Philip EPIQ7C (Philips Healthcare, Andover, USA) ultrasound that uses the X5-1 (1-5 MHz) or S8-3 (3-8 MHz) matrix-array transducers (Philips Healthcare, Andover, USA). LV interventricular or posterior wall thickness, internal diameter, volume in systole and diastole (IVSs, IVSd, LVPWs, LVPWd, LVIDs, LVIDd, ESV and EDV), ejection fraction (LVEF), and shorten fraction (LVFS) were measured. Relative wall thickness (RWT), LV mass (LVM), LVM index (LVMI), E/A ratio, Tei index, and global peak longitudinal strain (GLS) were measured as previously described [26].

Aortic annular diameter was measured. LV velocity time integral (VTI) was assessed by identifying the apical view in which peak flow velocity was maximal and, after calibration, tracing the black-white interface outlining the Doppler flow envelope. Aortic annular cross-sectional area was calculated as follows: π × (diameter/2)^2^. Doppler stroke volume (SV) was calculated as annular cross-sectional area multiplied by the VTI. Cardiac output (CO) was calculated as SV × HR. Total peripheral vascular resistance (TPVR) was calculated as MAP divided by CO [27]. The carotid intima-media thickness (cIMT), which was defined as the thickness of the intima and media of carotid artery, was averaged by measurements of 6 times at common carotid artery 1 cm below the carotid bulb on each side in 2-D images captured by C8-3 (3–8 MHz) or L15-7io (7–15 MHz) transducers [28, 29].

The sex-specific 95th percentiles (P95th) of LVMI (male: 33.76 g/m^2.7^ and female: 33.24 g/m^2.7^) were derived from our own cohort. LV hypertrophy (LVH) was defined as LVMI ≥ the sex-specific P95th of LVMI [26, 30]. All examinations were performed by experienced operators. Both the sonographers and the observers were blinded to the details of the participants.

### Statistical analysis

Comparisons of continuous variables were performed using the one-way analysis of variance (one-way ANOVA), followed by the Bonferroni post hoc test to adjust for multiple comparisons in different groups, when normality and homogeneity of variance assumptions are satisfied. Otherwise, the equivalent nonparametric tests were used. Both the Kolmogorov–Smirnov and Levene’s test were used to evaluate the normal distribution and homogeneity of variances. Categorical variables were compared using chi-square tests or Fisher’s exact tests.

Analysis of the associations of BW and GA with BP, LV structure, and function was conducted by constructing multi-factor linear regression models. To explore the independent effects of BW and GA on cardiovascular parameters, linear regression models were established in different BW and GA subgroups. Measurements for BW and GA were also used in the same model to analyze their interrelationship or mediated effect with each other. Odds ratios (OR) were calculated using a logistic regression model adjusted for the maternal and children’s factors. The interaction of BW and GA was analyzed in linear and logistic models. The additive interaction in logistic regression was assessed using the R package “epiR.” Restricted cubic spline models with 3 knots at the 5th, 50th, and 95th percentiles were constructed to determine the nonlinear correlation of BW or GA with cardiovascular measurements with “rms,” “splines,” and “ggplot2” R packages. Tests for nonlinearity were conducted by likelihood ratio tests.

Postnatal weight gain has been proven to play an important role in cardiovascular outcomes in preterm or LBW [31, 32]. BP may influence the LV structure and function (Additional file 1: Table S1). Weight gain from birth to 4 years old and the SBP of children were inputted into the regression models to examine their effect on cardiovascular parameters. For sensitivity analysis, multiple imputation was used to input the missing values in maternal information to analyze the relationship of BW and GA with blood pressure, LV structure, and function.

All statistical analysis were carried out using the SPSS 25.0 software program (IBM Corp., Armonk, NY, USA), Stata 15.0 (Stata Corporation, College Station, TX, USA), and R 4.0.4 (R Foundation for Statistical Computing). All tests were two-sided with a significance level of 0.05.

## Results

### The baseline characteristics

A total of 1194 mother–child pairs were included in the cardiovascular cohort in SBC. After excluding children due to missing information (*n* = 240) and birth defects (*n* = 11), a total of 943 (78.9%) children were included for final analysis (Additional file 1: Figure S1). In total, the study population consisted of 8.6% preterm (*n* = 81), 4.8% LBW (*n* = 45), and 7.6% macrosomia (*n* = 72) newborns. At 4 years of age, the difference in anthropometric indexes disappeared from GA groups but was maintained in the BW groups. LBW and preterm children gained more weight from birth to 4 years old than NBW and term (Table 1).

**Table 1:** Basic characteristics of participants

	LBW (*n* = 45)	NBW (*n* = 826)	Macrosomia (*n* = 72)	*P* value	Preterm (*n* = 81)	Term (*n* = 862)	*P* value	Total (*n* = 943)
**Maternal characteristics**								
Pre-pregnancy BMI, mean (SD), kg/m^2^	**22.34 (3.08)**	**21.54 (3.19)**	**22.65 (3.23)**	**0.01**	21.88 (3.20)	21.61 (3.18)	0.51	21.52 (3.03)
Maternal age, mean (SD), years	31.33 (3.32)	30.88 (3.51)	30.38 (3.56)	0.35	31.10 (4.12)	30.84 (3.47)	0.51	30.83 (3.52)
HDP, *N* (%)	5 (11.1%)	75 (9.1%)	4 (5.6%)	0.43	7 (8.6%)	77 (8.9%)	0.48	84 (8.9%)
Passive smoke, *N* (%)	12 (26.7%)	391 (47.3%)	32 (44.4%)	0.13	27 (33.3%)	406 (47.1%)	0.63	435 (46.1%)
Drink during pregnancy, *N* (%)	5 (11.1%)	127 (15.4%)	7 (9.7%)	0.82	10 (12.3%)	129 (15.0%)	0.86	139 (14.7%)
University background, *N* (%)	29 (64.4%)	690 (83.5%)	46 (63.9%)	0.51	51 (62.9%)	711 (82.5%)	0.41	765 (81.1%)
Ethic Han, *N* (%)	41 (91.1%)	826 (100%)	70 (97.2%)	0.52	75 (92.6%)	862 (100.0%)	0.28	937 (99.4%)
Income ≥ 100,000 yuan/year, *N* (%)	20 (44.4%)	459 (55.6%)	36 (50.0%)	0.29	35 (43.2%)	478 (55.4%)	0.66	515 (54.6%)
**Children characteristics**								
Birth height, mean (SD), cm	**46.84 (1.89)**	**49.75 (1.23)**	**51.60 (1.81)**	** < 0.001**	**47.95 (1.87)**	**49.89 (1.39)**	** < 0.001**	49.8 (1.50)
BW, mean (SD), kg	**2.19 (0.33)**	**3.31 (0.35)**	**4.23 (0.22)**	** < 0.001**	**2.52 (0.48)**	**3.39 (0.42)**	** < 0.001**	3.33 (0.48)
**BW groups, *N* (%)**								
LBW	/	/	/	/	**27 (33.3%)**	**18 (2.9%)**	** < 0.001**	45 (4.8%)
NBW					**54 (66.7%)**	**772 (89.6%)**	** < 0.001**	826 (87.6%)
MBW					**0 (0.0%)**	**72 (8.4%)**	** < 0.001**	72 (7.6%)
GA, mean (SD), weeks	**35.55 (2.79)**	**39.21** (**(1.41)**	**39.85 (0.79)**	** < 0.001**	**34.92 (2.15)**	**39.43 (1.05)**	** < 0.001**	39.13 (1.51)
**GA groups, *N* (%)**								
Preterm	**27 (60.0%)**	**54 (6.5%)**	**0 (0%)**	** < 0.001**	/	/	/	81 (8.6%)
Term	**18 (40.0%)**	**772 (93.5%)**	**72 (100%)**	** < 0.001**				862 (91.4%)
**Gender, *N* (%)**								
Boys	**23 (51.1%)**	**479 (58.0%)**	**50 (69.4%)**	**0.01**	32 (39.5%)	520 (54.1%)	0.13	552 (58.5%)
Girls	**22 (48.9%)**	**347 (42.0%)**	**22 (30.6%)**	**0.01**	49 (60.5%)	342 (39.7%)	0.13	391 (41.5%)
Height at 4, mean (SD),cm	**106.62 (4.33)**	**108.07 (4.81)**	**110.25 (4.62)**	** < 0.001**	107.91 (4.73)	107.24 (4.53)	0.71	107.26 (4.53)
Weight at 4, mean (SD), kg	**16.99 (2.08)**	**17.75 (2.83)**	**19.30 (3.08)**	** < 0.001**	17.50 (2.66)	17.27 (2.60)	0.70	17.28 (2.56)
BMI at 4, mean (SD) kg/m^2^	**14.93 (1.43)**	**15.13 (1.65)**	**15.83 (1.98)**	**0.002**	14.98 (1.66)	14.97 (1.59)	0.25	14.97 (1.59)
Postnatal weight gain, mean (SD), kg	**14.77 (2.15)**	**13.82 (2.42)**	**15.06 (3.01)**	** < 0.001**	**15.18 (2.61)**	**13.86 (2.45)**	** < 0.001**	13.94 (2.48)

The bold values were *P* < 0.05

*GA* Gestational age, *BW* Birthweight, *LBW* Low birthweight, *NBW* Normal birthweight, *BMI* Body mass index, *HDP* Hypertensive disorders in pregnancy, *GDM* Gestational diabetes mellitus

### BP, LV structure, and function differed between the GA and BW groups

In different BW groups, LVIDd, IVSs, LVIDs, LVM, SV, EDV, and ESV were smaller in the LBW group and larger in the macrosomia group than NBW. The SBP and LV function were not significantly different among BW groups. In different GA groups, the cardiovascular measurements were not significantly different in full term and preterm birth children (Table 2). After adjusting for maternal or children’s factors, the results were consistent (Additional file 1: Tables S2 and S3).

**Table 2:** The BP, LV structure, and function in different BW and GA groups

	LBW (*n* = 45)	NBW (*n* = 826)	Macrosomia (*n* = 72)	*P* value	Preterm (*n* = 81)	Term (*n* = 862)	*P* value	Total
								(*n* = 943)
**Blood pressure**								
SBP, mean (SD), mmHg	98.93 (7.93)	98.16 (7.67)	100.22 (7.24)	0.09	98.24 (8.17)	97.54 (7.72)	0.80	97.57 (7.72)
DBP, mean (SD), mmHg	57.63 (7.35)	57.46 (6.27)	57.20 (6.45)	0.94	57.72 (6.37)	57.04 (6.32)	0.89	57.04 (6.19)
MAP, mean (SD), mmHg	72.24 (6.65)	71.45 (5.98)	71.59 (5.61)	0.69	71.88 (6.55)	70.89 (5.99)	0.17	70.97 (5.95)
**LV structure**								
IVSd, mean (SD), mm	3.88 (0.63)	3.84 (0.53)	3.82 (0.31)	0.88	3.79 (0.33)	3.81 (0.59)	0.80	3.79 (0.56)
LVIDd, mean (SD), mm	**34.92 (2.44)**	**35.75 (2.55)**	**36.78 (2.20)**^**a,b**^	**0.001**	35.52 (2.72)	35.48 (2.53)	0.89	35.47 (2.51)
LVPWd, mean (SD), mm	4.02 (0.51)	4.14 (0.61)	4.22 (0.47)	0.28	4.15 (0.63)	4.15 (0.59)	10.00	4.14 (0.56)
IVSs, mean (SD), mm	**6.46 (0.86)**	**6.53 (0.98)**	**6.93 (0.93)**^**a**^	**0.006**	6.53 (0.88)	6.57 (0.97)	0.78	6.57 (0.95)
LVIDs, mean (SD), mm	**22.20 (1.78)**	**22.81 (1.97)**	**23.43 (2.10)**^**a,b**^	**0.01**	22.69 (1.94)	22.57 (1.96)	0.63	22.57 (1.94)
LVPWs, mean (SD), mm	7.82 (0.93)	7.80 (0.99)	8.03 (0.95)	0.18	7.85 (0.90)	7.87 (0.95)	0.84	7.84 (0.91)
RWT	0.23 (0.04)	0.23 (0.04)	0.22 (0.02)	0.35	0.23 (0.03)	0.23 (0.04)	0.92	0.20 (0.10)
LVM, mean (SD), g	**30.44 (4.66)**	**32.34 (6.38)**	**34.47 (5.55)**^**a,b**^	**0.006**	31.62 (5.09)	31.83 (6.33)	0.79	31.66 (6.04)
LVMI, mean (SD), g/m^2.7^	25.58 (3.57)	26.19 (4.83)	26.30 (3.74)	0.75	25.73 (4.03)	26.34 (4.97)	0.32	26.20 (4.74)
cIMT, mean (SD), *10^−2^mm	41.35 (4.84)	40.45 (4.58)	40.82 (4.59)	0.58	40.88 (4.76)	40.76 (4.81)	0.87	40.78 (4.81)
**LV function**								
LVEF, mean (SD), %	66.99 (4.27)	69.21 (4.08)	66.93 (4.42)	0.97	66.54 (3.98)	66.93 (4.12)	0.46	66.90 (4.14)
LVFS, mean (SD), %	36.37 (3.37)	36.17 (3.21)	36.50 (3.49)	0.70	3 6.06 (3.12)	36.36 (3.23)	0.47	36.34 (3.25)
Tei index, mean (SD), %	44.82 (6.44)	43.77 (7.03)	43.01 (6.17)	0.45	44.20 (7.85)	43.22 (6.27)	0.21	43.26 (6.20)
E/A	1.78 (0.35)	1.81 (0.36)	1.79 (0.39)	0.71	1.81 (0.30)	1.80 (0.34)	0.79	1.81 (0.40)
CO, mean (SD), L/min	4.09 (1.01)	4.22 (1.20)	4.55 (1.39)	0.10	4.37 (1.27)	4.19 (1.13)	0.22	4.19 (1.16)
SV, mean (SD), mL	**43.37 (10.32)**	**46.36 (12.98)**	**51.13 (14.94)**^**a,b**^	**0.008**	47.89 (12.87)	45.70 (12.41)	0.16	45.71 (12.56)
TPVR, mean (SD), dyne^*^s/cm^5^	1540.19 (525.32)	1465.55 (447.13)	1386.20 (491.63)	0.28	1427.89 (455.43)	1463.51 (444.36)	0.54	1469.40 (450.46)
EDV, mean (SD), %	**50.97 (8.80)**	**53.94 (9.23)**	**57.65 (8.26)**^**a,b**^	**0.001**	53.22 (9.85)	53.00 (8.98)	0.85	52.95 (8.89)
ESV, mean (SD), %	**16.77 (3.50)**	**17.99 (3.85)**	**19.22 (4.11)**^**a,b**^	**0.008**	17.76 (3.84)	17.52 (3.77)	0.61	17.50 (3.75)
VTI, mean (SD), cm	21.72 (3.63)	21.50 (3.24)	21.74 (3.93)	0.80	22.31 (3.51)	21.52 (3.25)	0.05	21.57 (3.28)
GLS, mean (SD), %	23.49 (2.27)	23.57 (2.30)	23.36 (2.33)	0.87	23.39 (2.62)	23.58 (2.28)	0.65	23.54 (2.26)
Prehypertension, *N* (%)	4 (8.9%)	131 (15.8%)	6 (8.3%)	0.13	11 (13.6%)	130 (15.1%)	0.09	141 (14.9%)
Hypertension, *N* (%)	6 (13.3%)	85 (10.3%)	10 (13.9%)	0.71	11 (13.6%)	89 (10.3%)	0.85	101 (10.7%)
LVH, *N* (%)	0 (0.0%)	53 (6.4%)	2 (2.8%)	0.27	2 (2.5%)	53 (6.1%)	0.57	55 (5.8%)

The bold values were *P* < 0.05

*LBW* Low birthweight, *NBW* Normal birthweight, *SBP* Systolic blood pressure, *DBP* Diastolic blood pressure, *LV* Left ventricle, *IVSd* Ventricle interventricular septal thickness in diastole, *LVIDd* LV internal diameter in diastole, *LVPWd* LV posterior wall thickness in diastole, *IVSs* Ventricle interventricular septal thickness in systole, *LVIDs* LV internal diameter in systole, *LVPWs* LV posterior wall thickness in systole, *RWT* Relative wall thickness, *LVM* Left ventricle mass, *LVMI* LV mass index, *cIMT* carotid artery intima-media thickness, *LVEF* LV ejection fraction, *LVFS* LV fractional shorting, *CO* Cardiac output, *SV* Stroke volume, *TPVR* Total peripheral vascular resistance, *MAP* Mean arterial pressure, *EDV* End diastolic volume, *ESV* End systolic volume, *VTI* Velocity time integral, *GLS* Global longitudinal strain, *LVH* LV hypertrophy

^a^Compared with NBW were significant using Bonferroni test

^b^Compared with LBW were significant using Bonferroni test

### The different effect of GA and BW on BP

GA had a significant negative correlation with SBP [*β* =  − 0.41, 95% CI: (− 0.76, − 0.07)] and MAP [*β* =  − 0.36, 95% CI: (− 0.66, − 0.07)], but not with DBP after adjusting for maternal and children’s factors (Table 3). The risk of prehypertension decreased with increasing GA [OR = 0.54, 95% CI: (0.32, 0.93)] after adjusting for BMI (Table 4). BMI is a key negative confounder in GA and BP. After adjusting for postnatal weight gain, the significant relationship between BP and GA disappeared, suggesting postnatal weight gain was a completed mediator in GA and SBP (Additional file 1: Table S4).

**Table 3:** The independent and interaction effect of BW and GA on BP, LV structure, and function

	BW			GA			
	Crude Model	Model 1	Model 2	Crude Model	Model 1	Model 2	*P* for interaction^#^
**Blood pressure**							
SBP (mmHg)	0.79 (− 0.24, 1.81)	− 0.03 (− 1.31, 1.24)	− 0.77 (− 2.02, 0.48)	** − 0.30 (− 0.58, − 0.02)**	** − 0.47 (− 0.84, − 0.11)**	** − 0.41 (− 0.76, − 0.07)**	0.63
DBP (mmHg)	− 0.47 (− 1.29, 0.36)	− 0.29 (− 1.39, 0.81)	− 0.59 (− 1.71, 0.52)	− 0.15 (− 0.38, 0.08)	− 0.17 (− 0.49, 0.15)	− 0.16 (− 0.48, 0.16)	0.97
MAP (mmHg)	− 0.22 (− 1.00, 0.56)	− 0.51 (− 1.54, 0.52)	− 0.95 (− 1.99, 0.08)	− 0.21 (− 0.43, 0.01)	** − 0.38 (− 0.67, − 0.08)**	** − 0.36 (− 0.66, − 0.07)**	0.98
**LV structure**							
IVSd (mm)	0.04 (− 0.03, 0.12)	0.03 (− 0.06, 0.12)	0.02 (− 0.07, 0.12)	− 0.01 (− 0.03, 0.01)	− 0.02 (− 0.05, 0.01)	− 0.41 (− 0.92, 0.11)	0.51
LVIDd (mm)	**1.04 (0.71, 1.38)**	**1.02 (0.61, 1.43)**	**0.78 (0.37, 1.19)**	**0.11 (0.01, 0.20)**	0.11 (− 0.01, 0.23)	− 0.02 (− 0.05, 0.01)	**0.01**
LVPWd (mm)	**0.13 (0.54, 0.21)**	**0.11 (0.01, 0.21)**	0.08 (− 0.02, 0.18)	− 0.01 (− 0.03, 0.01)	− 0.16 (− 0.05, 0.01)	− 0.02 (− 0.04, 0.01)	0.97
IVSs (mm)	**0.31 (0.18, 0.44)**	**0.24 (0.09, 0.40)**	**0.18 (0.02, 0.34)**	0.02 (− 0.02, 0.05)	0.01 (− 0.03, 0.06)	0.01 (− 0.03, 0.06)	0.90
LVIDs (mm)	**0.71 (0.44, 0.97)**	**0.93 (0.61, 1.26)**	**0.79 (0.46, 1.11)**	0.05 (− 0.02, 0.13)	**0.10 (0.00, 0.19)**	**0.10 (0.00, 0.19)**	0.08
LVPWs (mm)	**0.24 (0.12, 0.36)**	0.12 (− 0.04, 0.28)	0.03 (− 0.13, 0.19)	0.00 (− 0.03, 0.04)	− 0.01 (− 0.06, 0.04)	− 0.01 (− 0.05, 0.04)	0.18
RWT	0.00 (− 0.01, 0.01)	0.00 (− 0.01, 0.01)	0.00 (− 0.01, 0.01)	0.00 (0.00, 0.00)	0.00 (0.00, 0.00)	0.00 (0.00, 0.00)	0.31
LVM (g)	**2.58 (1.78, 3.39)**	**2.44 (1.42, 3.45)**	**1.88 (0.87, 2.90)**	0.09 (− 0.15, 0.33)	0.00 (− 0.30, 0.31)	0.01 (− 0.28, 0.30)	**0.05**
LVMI (g/cm^2.7^)	**0.66 (0.01, 1.30)**	0.59 (− 0.26, 1.25)	0.15 (− 0.69, 1.00)	0.09 (− 0.10, 0.28)	0.09 (− 0.15, 0.33)	0.09 (− 0.15, 0.33)	0.67
cIMT (*10^−2^ mm)	0.28 (− 0.53, 1.09)	0.04 (− 1.00, 1.09)	0.08 (− 0.99, 1.15)	0.11 (− 0.11, 0.32)	0.16 (− 0.12, 0.45)	0.16 (− 0.12, 0.45)	0.51
**LV function**							
LVEF (%)	− 0.15 (− 0.71, 0.42)	** − 1.00 (− 1.73, − 0.28)**	** − 1.02 (− 1.76, − 0.27)**	0.05 (− 0.11, 0.20)	− 0.13 (− 0.34, 0.08)	− 0.13 (− 0.34, 0.08)	0.74
LVFS (%)	− 0.03 (− 0.47, 0.42)	** − 0.71 (− 1.28, − 0.14)**	** − 0.72 (− 1.31, − 0.14)**	0.04 (− 0.08, 0.17)	− 0.10 (− 0.26, 0.07)	− 0.10 (− 0.26, 0.07)	0.61
Tei index (%)	− 0.23 (− 1.06, 0.60)	0.03 (− 1.05, 1.12)	0.16 (− 0.94, 1.26)	− 0.12 (− 0.36, 0.11)	0.03 (− 0.28, 0.34)	0.03 (− 0.28, 0.34)	0.63
E/A	0.00 (− 0.04, 0.04)	0.01 (− 0.05, 0.06)	0.01 (− 0.04, 0.07)	0.00 (− 0.01, 0.02)	0.01 (− 0.01, 0.26)	0.01 (− 0.01, 0.03)	0.82
CO (L/min)	**0.24 (0.08, 0.40)**	**0.24 (0.04, 0.44)**	0.15 (− 0.06, 0.35)	− 0.01 (− 0.06, 0.03)	− 0.02 (− 0.08, 0.04)	− 0.01 (− 0.07, 0.04)	0.53
SV (ml)	**3.91 (2.21, 5.61)**	**4.35 (2.19, 6.51)**	**3.40 (1.23, 5.56)**	0.07 (− 0.40, 0.54)	0.10 (− 0.52, 0.71)	0.11 (− 0.49, 0.72)	0.80
TPVR (dynes*s/cm^5^)	** − 77.19 (− 141.11, − 13.26)**	** − 93.15 (− 177.34, − 8.96)**	− 75.53 (− 160.79, 9.74)	6.17 (− 11.09, 23.48)	3.85 (− 20.19, 27.88)	2.87 (− 21.03, 26.77)	0.53
EDV (ml)	**3.74 (2.56, 4.93)**	**3.72(2.24, 5.19)**	**2.86 (1.39, 4.32)**	**0.36 (0.01, 0.70)**	0.39 (− 0.04, 0.83)	0.40 (− 0.02, 0.81)	**0.009**
ESV (ml)	**1.38 (0.87, 1.88)**	**1.80 (1.17, 2.44)**	**1.51 (0.87, 2.15)**	0.09 (− 0.05, 0.23)	**0.19 (0.00, 0.38)**	**0.19 (0.01, 0.37)**	0.07
VTI (cm)	0.02 (− 0.43, 0.46)	0.11 (− 0.48, 0.69)	− 0.08 (− 0.67, 0.51)	− 0.05 (− 0.17, 0.08)	− 0.04 (− 0.21, 0.12)	0.04 (− 0.21, 0.12)	1.00
GLS (%)	− 0.15 (− 0.56, 0.26)	− 0.40 (− 0.94, 0.15)	− 0.32 (− 0.87, 0.23)	0.07 (− 0.05, 0.18)	− 0.01 (− 0.16, 0.15)	− 0.01 (− 0.17, 0.15)	0.09

The data were presented as *β* (95% CI) in linear regression models. The missing values were not inputted (crude model: *n* = 943, model 1: *n* = 786, model 2: *n* = 583)

The bold values were *P* < 0.05

Model 1: adjusted for maternal nationality, scholarship, income, HDP, GDM, drink history, passive smoke history, and gender of children

Model 2: model 1 + BMI at 4 years old

*BW* Birthweight, *GA* Gestational age, *SBP* Systolic blood pressure, *DBP* Diastolic blood pressure, *LV* Left ventricle, *IVSd* Ventricle interventricular septal thickness in diastole, *LVIDd* LV internal diameter in diastole, *LVPWd* LV posterior wall thickness in diastole, *IVSs* Ventricle interventricular septal thickness in systole, *LVIDs* LV internal diameter in systole, *LVPWs* LV posterior wall thickness in systole, *RWT* Relative wall thickness, *LVM* Left ventricle mass, *LVMI* LV mass index, *cIMT* carotid artery intima-media thickness, *LVEF* LV ejection fraction, *LVFS* LV fractional shorting, *CO* Cardiac output, *SV* Stroke volume, *TPVR* Total peripheral vascular resistance, *MAP* Mean arterial pressure, *EDV* End diastolic volume, *ESV* End systolic volume, *VTI* Velocity time integral, *GLS* Global longitudinal strain, *BMI* Body mass index, *HDP* Hypertensive disorders in pregnancy,*GDM* Gestational diabetes mellitus

^#^Adjusted for maternal nationality, scholarship, income, HDP, GDM, drink history, passive smoking, gender of children, and BMI at 4

**Table 4:** The cardiovascular risk with GA and BW

	Prehypertension (*n* = 141)			Hypertension (*n* = 101)			LVH (*n* = 55)		
	Crude	Model 1	Model 2	Crude	Model 1	Model 2	Crude	Model 1	Model 2
BW	0.99 (0.89, 1.10)	0.90 (0.79, 1.03)	0.92 (0.80, 1.05)	0.93 (0.83, 1.05)	0.92 (0.79, 1.07)	0.94 (0.80, 1.10)	1.60 (0.84, 3.04)	1.00 (1.00, 1.00)	1.59 (0.68, 3.73)
GA	0.76 (0.50, 1.13)	0.59 (0.35, 1.00)	**0.54 (0.32 0.93)**	1.16 (0.70, 1.76)	1.02 (0.57, 1.84)	0.80 (0.44, 1.45)	1.10 (0.91, 1.33)	1.05 (0.82, 1.33)	1.05 (0.82, 1.34)

Data were presented as OR (95% CI). *P* for multiplicative and addictive interaction of BW and GA were not significant. The missing values were not inputted (crude model: *n* = 943, model 1: *n* = 786, model 2: *n* = 583)

The bold values were *P* < 0.05

Model 1: adjusted for maternal nationality, scholarship, income, HDP, GDM, drink history, passive smoke history, and gender of children

Model 2: model 1 + BMI at 4 years old

The 90th and 95th percentile of SBP and DBP for the sex and height were defined according to the Chinese standard [24]. Prehypertension was defined as P95th > SBP and/or DBP ≥ P90th. Hypertension was defined as SBP and/or DBP ≥ P95th. LV hypertrophy (LVH) was defined as LVMI ≥ the sex-specific P95th of LVMI

*BW* Birthweight, *GA* Gestational age, *LVH* Left ventricle hypertrophy, *HDP* Hypertensive disorders in pregnancy, *GDM* Gestational diabetes mellitus

**Fig. 1:**
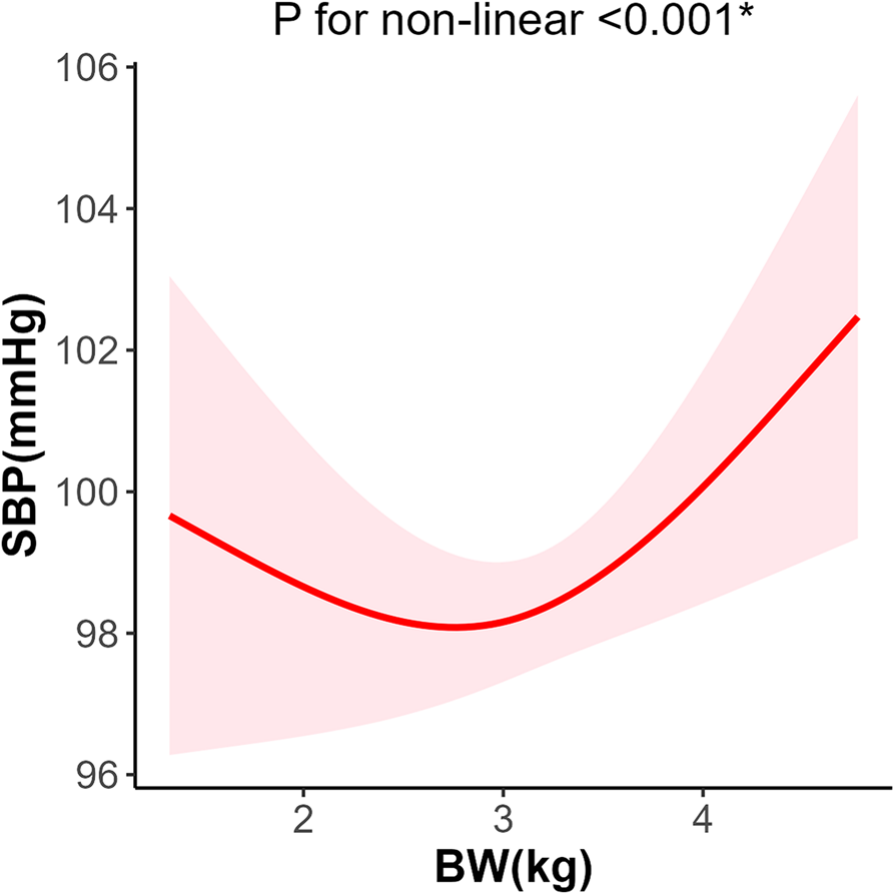
The restricted cubic spline of BW and SBP (*n* = 943). The red lines indicate the predicted cardiovascular parameters derived from the restricted cubic spline regression model with 3 knots at the 5th, 50th, and 95th percentiles of BW. The shadow indicates the 95%CIs. Tests for non-linearity were conducted by using likelihood ratio tests. BW, birthweight; SBP, systolic blood pressure

The BW was found not to have a significant association with BP or hypertension in either the linear or the logistic regression models (Tables 3 and 4). However, a non-linear association between BP and BW was found. The smoothing line of BW and SBP was in a U-shape (*P* for non-linear < 0.001) (Fig. 1).

The positive linear correlation between BW and GA was strong [*β* = 0.18, 95% CI: (0.16, 0.19)], though GA still influenced the BP independently from BW. When putting BW and GA in the same model, the SBP remained negatively correlated with GA but not with BW (Additional file 1: Table S5). In different BW groups, SBP was negatively associated with GA in participants with preterm birth children, including LBW [*β* =  − 0.58, 95% CI: (− 1.13, − 0.04)] and NBW groups [*β* =  − 0.55, 95% CI: (− 1.04, − 0.07)]). In different GA subgroups, the BW had a negative correlation with SBP only in preterm children not in term [*β* =  − 4.22, 95% CI: (− 8.02, − 0.42)] (Additional file 1: Table S6). Preterm birth played a more important role in the occurrence of high BP than LBW.

### The different effect of GA and BW on LV structure and function

BW was found to be associated with higher LV wall thickness [IVSs: *β* = 0.18, 95% CI:(0.02,0.34)], interventricular diameters [LVIDd: *β* = 0.78, 95% CI:(0.37,1.19), LVIDs: *β* = 0.79, 95% CI:(0.46, 1.11)], volume [EDV: *β* = 2.86, 95% CI:(1.39,4.32), ESV: *β* = 1.51, 95% CI:(0.87, 2.15)], and LVM [*β* = 1.88, 95% CI:(0.87, 2.90)]. LV function indexes of LVEF [*β* =  − 1.02, 95% CI: (− 1.76, − 0.27)] and LVFS [*β* =  − 0.72, 95% CI: (− 1.31, − 0.14)] decreased with increasing BW following adjustment for confounders (Table 3). These results were stable in non-linear regression models (Additional file 1: Figure S2), though the risk of LVH was not significant (Table 4). In the linear regression or non-linear models, the correlation of LV structure and function with GA was not significant (Table 3 and Additional file 1: Figure S2).

BW influenced the LV structure and function independently from GA. When inputting BW and GA into the same models (Additional file 1: Table S5), the effect of BW on the LV structure and function were not changed, even after adjusting for the postnatal weight gain and SBP (Additional file 1: Table S4). However, the GA was found to have a negative correlation with LVPWd, CO, SV, and LVM (Additional file 1: Table S5). After adjusting for postnatal weight gain and SBP, the significance of the relationship between GA and these indexes disappeared (Additional file 1: Table S4). In different GA groups, the effect of BW on the LV structure and function were stable in full term children. GA had no significant association with LV structure and function in any of the BW subgroups (Additional file 1: Table S6). In sensitivity analysis, the results were stable (Additional file 1: Table S7-S8).

## Discussion

To the best of our knowledge, this study is the first to suggest that GA and BW have different effects on the cardiovascular system, though with a strong association with each other. GA mainly influenced the BP while BW had an independent impact on cardiac structure and function. The relationship between BW and BP was in a U-shape, and the interaction effect of GA and BW on cardiovascular parameters was not significant.

This study suggested that GA had a negative association with BP and low GA increased the risk of prehypertension, even in early childhood. The risk of hypertension was not significant associated with GA, potentially because of the small sample size of preterm children with hypertension in our population. Recent studies have also found a significant inverse correlation between SBP and GA [3, 16, 33], and individuals of preterm birth or those with fetal growth restriction have a tendency for elevated BP levels in childhood and adulthood [34–36]. Some studies found that LBW might increase the risk of hypertension in the future [37, 38], but LBW individuals are usually preterm. However, no study has explored the independent association of GA and BW on BP. Our results suggest that for LBW children, the increase in BP maybe arise independently from preterm birth. Increased BW for preterm birth infants could potentially reduce BP in childhood.

BP is mainly influenced by BMI [39]. Obese children usually have higher BP which could explain the high BW children have higher BP than normal BW in the U-shape line of BW and BP. For preterm or LBW children, the BMI was lower than normal; however, they may still have higher BP and risk of hypertension during adolescence and adulthood [40–42]. This phenomenon is particularly interesting. It can be hypothesized that the mechanism is as follows: GA mainly influences children by the duration of the intra or extra uterine environmental exposure and affects the degree of fetal organ development through perinatal reprogramming [43]. It is determined by the maternal and/or fetal health condition. High SBP and normal or low DBP indicates high pulse pressure and increased blood vessel stiffness. Preterm and fetal growth restriction babies have defects in renal development with less nephron [44]. Vascular or cardiac development defection or early exposure to extra uterine environment can cause early hypoxic or inflammation damage, which then leads to accelerated vascular aging, vascular elastance reduction, and cardiac remodeling [40–42]. Additionally, the sympathetic system is overactivated, and parasympathetic nervous system tone is deficient in preterm infants [33]. Early stress response stimulates elevation of BP raising hormones that finally contribute to a greater SBP [43]. Furthermore, higher BP in preterm birth children has been proposed to be influenced by other intrauterine or postnatal factors, aside from their own BMI state, such as maternal disease, like hypertension or diabetes, and children’s early fast weight gain or catch-up growth pattern [45]. In this study, after adjusting for BMI, the risk of low GA with prehypertension was significant. Postnatal weight gain played a mediating effect on GA and BP, suggesting postnatal growth might play an important role in the relationship between GA and BP. For preterm or LBW, early overnutrition can improve malnutrition or growth retardation, but it increases the risk of CVD in the future [46–48]. Early growth management in preterm infants remains a challenge and is worthy of further exploration.

Recent studies have found similar results of a relationship between BW and LV structure and function. The studies by Toemen et al. [49] and Zhang et al. [50] found that children who are larger at birth, and have a longtime burden of excessive growth, are at increased risk of LV hypertrophy in adolescence and adulthood. A study by Harris et al. [51] similarly found that very low BW adults had smaller LVs, higher LV elastance, and lower arterial elastance. However, existing studies usually focus on specific disease participants and have only addressed either GA or BW with cardiovascular measurements. Our study is the first to report an independent and interactive association of GA and BW on cardiovascular measurements in continuous data.

BW influences LV structure and function potentially via BMI burden from birth. BW reflects the size and growth condition of body and organs during the fetal stage. It is influenced by GA, maternal nutrition state, intrauterine environment, fetal organ weight, and so on. However, both BP and LV structure are determined to some degree by the growth and BMI of children. The number of fat cells has been determined previously in neonates [52]. The body or organ size in the future is determined by the size in early life [53]. It is known that high BW may contribute to maintenance of BMI burden throughout life [22, 54]. BP and LV wall thickness are known to have a positive association with BMI [39]. Excessive BMI can be detrimental to LV function and increase the risk of LV dysfunction, hypertrophy, and hypertension in both adolescents and adults [19, 55–58]. However, in early childhood, changes in LV structure may arise from physiological factors rather than pathological ones. Function damage would be relatively small while the heart would get bigger and heavier as the body grows. Therefore, the growth of the body is typically faster than the growth of the heart. LVMI therefore might not change significantly with BW while only increases in LVM are observed.

In other studies, GA has been found to influence both cardiac structure and function. Preterm children might have defects in both cardiac development and growth. Goss et al. [10] and Mohlkert et al. [17] found that children, adolescents, and young adults born prematurely had significantly smaller biventricular cardiac chamber size and decreased cardiac mass with altered systolic and diastolic functions. But, in our study, the cardiac structure and function were not influenced by GA. The reason might be like this. GA had a negative association with BP, which means that preterm babies might have a higher BP in the future. It has been proposed that LV hypertrophy is caused by high SBP [59]. According to current studies, preterm children have smaller heart sizes and masses than term babies. For preterm children, BP and the influence of postnatal catch-up growth on cardiac growth could balance the change of cardiac structure in the early childhood. However, if the high BP condition is persistent, cardiac hypertrophy might occur in the future [6].

It should be noted that this study had some limitations. First, the sample size of extreme preterm and post-term birth and extremely low or macrosomia children were relatively small in our study, which may have contributed to bias to the results. Further studies with larger sample sizes are needed to draw stronger conclusions. Second, echocardiography for younger children requires sedatives to be administered during the examination, and so it was only performed at 4 years old, presenting a major study limitation as these parameters may have changed during early development. As this is an ongoing cohort, longitudinal assessment of cardiovascular parameters will be performed again in a further follow-up. Third, we did not collect blood metabolism biomarkers, other cardiovascular measurements, and information of other confounders, such as daily exercise, diet, and sleep. Further studies with more detailed information are recommended.

## Conclusions

GA and BW have different effects on cardiovascular measurements in 4 years old. BP was negatively correlated with GA. The relationship of BW and BP was non-linear. BW had a greater impact on LV structure and function than GA. The LV wall thickness, interventricular diameter, volume, and mass increased with BW. The LV function decreased with BW. Small GA played a more important role in the occurrence of high BP than low or high BW. Heart size and function at 4 years old was influenced mostly by BW and not by GA. BW and GA are important factors in early childhood cardiovascular health. Therefore, BW control, prevention of preterm birth, and early cardiovascular risk screening in children are recommended to help early prevention on children from getting cardiovascular diseases.

## Supplementary Information

**Additional file 1: Figure S1.** The flowchart of the study. **Figure S2.** The restricted cubic spline of BW and GA with cardiovascular parameters. **Table S1.** The influence of SBP and DBP on LV structure and function. **Table S2-S3.** The BP, LV structure and function in different BW and GA groups in linear regression models. **Table S4.** The association of BW and GA with BP, LV structure and function after adjusted for children weight gain from birth and BP. **Table S5.** The effect of BW and GA on BP, LV structure and function by putting them in the same models. **Table S6.** The association of BW and GA with BP, LV structure and function in fixed GA or BW groups. **Table S7-S8.** The effect of BW and GA on BP, LV structure and function after multiple imputation.

## Data Availability

Deidentified individual participant data (including data dictionaries) will be made available, in addition to study protocols, the statistical analysis plan, and the informed consent form. The data will be made available upon publication to researchers who provide a methodologically sound proposal for use in achieving the goals of the approved proposal. Proposals should be submitted to wangjian@xinhuamed.com.cn.

## Abbreviations

GA: Gestational age
BW: Birthweight
LBW: Low birthweight
NBW: Normal birthweight
BMI: Body mass index
HDP: Hypertensive disorders in pregnancy
GDM: Gestational diabetes mellitus
SBP: Systolic blood pressure
DBP: Diastolic blood pressure
LV: Left ventricle
IVSd: Ventricle interventricular septal thickness in diastole
LVIDd: LV internal diameter in diastole
LVPWd: LV posterior wall thickness in diastole
IVSs: Ventricle interventricular septal thickness in systole
LVIDs: LV internal diameter in systole
LVPWs: LV posterior wall thickness in systole
RWT: Relative wall thickness
LVM: Left ventricle mass
LVMI: LV mass index
cIMT: Carotid artery intima-media thickness
LVEF: LV ejection fraction
LVFS: LV fractional shorting
CO: Cardiac output
SV: Stroke volume
TPVR: Total peripheral vascular resistance
MAP: Mean arterial pressure
EDV: End diastolic volume
ESV: End systolic volume
VTI: Velocity time integral
GLS: Global longitudinal strain

## References

[1] Barker DJ, Winter PD, Osmond C, Margetts B, Simmonds SJ (1989). Weight in infancy and death from ischaemic heart disease. Lancet.

[2] Lynch J, Smith GD (2005). A life course approach to chronic disease epidemiology. Annu Rev Public Health.

[3] de Jong F, Monuteaux MC, van Elburg RM, Gillman MW, Belfort MB (2012). Systematic review and meta-analysis of preterm birth and later systolic blood pressure. Hypertension.

[4] Wang X, Dong Y, Zou Z, Ma J, Yang Z, Gao D, et al. Low birthweight is associated with higher risk of high blood pressure in Chinese girls: results from a national cross-sectional study in China. Int J Environ Res Public Health. 2019;16(16):2898. 10.3390/ijerph16162898 PMC6718998 31412652

[5] Carr H, Cnattingius S, Granath F, Ludvigsson JF, Edstedt Bonamy AK (2017). Preterm birth and risk of heart failure up to early adulthood. J Am Coll Cardiol.

[6] Lewandowski AJ, Augustine D, Lamata P, Davis EF, Lazdam M, Francis J (2013). Preterm heart in adult life: cardiovascular magnetic resonance reveals distinct differences in left ventricular mass, geometry, and function. Circulation.

[7] Crump C, Howell EA, Stroustrup A, McLaughlin MA, Sundquist J, Sundquist K (2019). Association of preterm birth with risk of ischemic heart disease in adulthood. JAMA Pediatr.

[8] Lawlor DA, Ronalds G, Clark H, Smith GD, Leon DA (2005). Birth weight is inversely associated with incident coronary heart disease and stroke among individuals born in the 1950s: findings from the Aberdeen Children of the 1950s prospective cohort study. Circulation.

[9] Larsson SC, Drca N, Jensen-Urstad M, Wolk A (2015). Incidence of atrial fibrillation in relation to birth weight and preterm birth. Int J Cardiol.

[10] Goss KN, Haraldsdottir K, Beshish AG, Barton GP, Watson AM, Palta M (2020). Association between preterm birth and arrested cardiac growth in adolescents and young adults. JAMA Cardiol.

[11] Fattal-Valevski A, Bassan H, Bernheim J, Redianu B, Leitner Y, Harel S (2011). Blood pressure values in 8–12 year old children with a history of intrauterine growth retardation. Isr Med Assoc J.

[12] Geserick M, Vogel M, Gausche R, Lipek T, Spielau U, Keller E (2018). Acceleration of BMI in early childhood and risk of sustained obesity. N Engl J Med.

[13] Renom Espineira A, Fernandes-Rosa FL, Bueno AC, de Souza RM, Moreira AC, de Castro M (2011). Postnatal growth and cardiometabolic profile in young adults born large for gestational age. Clin Endocrinol (Oxf).

[14] Wang H, Du B, Wu Y, Li Z, Niu Y, Ouyang F (2021). Sex-disparity in the association between birthweight and cardiovascular parameters in 4-year-old children: a Chinese cohort study. Front Nutr.

[15] Hardy R, Kuh D, Langenberg C, Wadsworth ME (2003). Birthweight, childhood social class, and change in adult blood pressure in the 1946 British birth cohort. Lancet.

[16] Lawlor DA, Hübinette A, Tynelius P, Leon DA, Smith GD, Rasmussen F (2007). Associations of gestational age and intrauterine growth with systolic blood pressure in a family-based study of 386,485 men in 331,089 families. Circulation.

[17] Mohlkert LA, Hallberg J, Broberg O, Rydberg A, Halvorsen CP, Liuba P, et al. The preterm heart in childhood: left ventricular structure, geometry, and function assessed by echocardiography in 6-year-old survivors of periviable births. J Am Heart Assoc. 2018;7(2):e007742. 10.1161/JAHA.117.007742 PMC5850168 29353231

[18] Edvardsson VO, Steinthorsdottir SD, Eliasdottir SB, Indridason OS, Palsson R (2012). Birth weight and childhood blood pressure. Curr Hypertens Rep.

[19] Hardy R, Ghosh AK, Deanfield J, Kuh D, Hughes AD (2016). Birthweight, childhood growth and left ventricular structure at age 60–64 years in a British birth cohort study. Int J Epidemiol.

[20] Lurbe E, Ingelfinger J (2021). Developmental and early life origins of cardiometabolic risk factors: novel findings and implications. Hypertension.

[21] Juonala M, Cheung MM, Sabin MA, Burgner D, Skilton MR, Kähönen M (2015). Effect of birth weight on life-course blood pressure levels among children born premature: the Cardiovascular Risk in Young Finns Study. J Hypertens.

[22] Zhao Y, Wang SF, Mu M, Sheng J (2012). Birth weight and overweight/obesity in adults: a meta-analysis. Eur J Pediatr.

[23] Zhang J, Tian Y, Wang W, Ouyang F, Xu J, Yu X (2019). Cohort profile: the Shanghai Birth Cohort. Int J Epidemiol..

[24] Hui F, Yinkun Y, Jie Mi (2017). Blood pressure reference standard for Chinese children aged 3–17 by sex, age and height. Zhonghua Gao Xue Ya Za Zhi.

[25] Lai WW, Geva T, Shirali GS, Frommelt PC, Humes RA, Brook MM (2006). Guidelines and standards for performance of a pediatric echocardiogram: a report from the Task Force of the Pediatric Council of the American Society of Echocardiography. J Am Soc Echocardiogr.

[26] Wang J, Du B, Wu Y, Li Z, Chen Q, Zhang X (2021). Association of maternal gestational weight gain with left ventricle geometry and function in offspring at 4 years of age: a prospective birth cohort study. Front Pediatr.

[27] Drukteinis JS, Roman MJ, Fabsitz RR, Lee ET, Best LG, Russell M (2007). Cardiac and systemic hemodynamic characteristics of hypertension and prehypertension in adolescents and young adults: the Strong Heart Study. Circulation.

[28] Cobble M, Bale B (2010). Carotid intima-media thickness: knowledge and application to everyday practice. Postgrad Med.

[29] Naqvi TZ, Lee MS (2014). Carotid intima-media thickness and plaque in cardiovascular risk assessment. JACC Cardiovasc Imaging.

[30] Ganau A, Devereux RB, Roman MJ, de Simone G, Pickering TG, Saba PS (1992). Patterns of left ventricular hypertrophy and geometric remodeling in essential hypertension. J Am Coll Cardiol.

[31] Bowers K, Liu G, Wang P, Ye T, Tian Z, Liu E (2011). Birth weight, postnatal weight change, and risk for high blood pressure among Chinese children. Pediatrics.

[32] Lule SA, Namara B, Akurut H, Muhangi L, Lubyayi L, Nampijja M (2019). Are birthweight and postnatal weight gain in childhood associated with blood pressure in early adolescence? Results from a Ugandan birth cohort. Int J Epidemiol.

[33] Sutherland MR, Bertagnolli M, Lukaszewski MA, Huyard F, Yzydorczyk C, Luu TM (2014). Preterm birth and hypertension risk: the oxidative stress paradigm. Hypertension.

[34] Lewington S, Clarke R, Qizilbash N, Peto R, Collins R (2002). Age-specific relevance of usual blood pressure to vascular mortality: a meta-analysis of individual data for one million adults in 61 prospective studies. Lancet.

[35] Rotteveel J, Twisk JW, van Weissenbruch MM, Delemarre-Van de Waal HA (2008). Infant and childhood growth patterns, insulin sensitivity, and blood pressure in prematurely born young adults. Pediatrics..

[36] Willemsen RH, de Kort SW, van der Kaay DC, Hokken-Koelega AC (2008). Independent effects of prematurity on metabolic and cardiovascular risk factors in short small-for-gestational-age children. J Clin Endocrinol Metab.

[37] Kanda T, Murai-Takeda A, Kawabe H, Itoh H (2020). Low birth weight trends: possible impacts on the prevalences of hypertension and chronic kidney disease. Hypertens Res.

[38] Huxley R, Neil A, Collins R (2002). Unravelling the fetal origins hypothesis: is there really an inverse association between birthweight and subsequent blood pressure?. Lancet.

[39] Liu Y, Yan Y, Jiang T, Li S, Guo Y, Fernandez C (2020). Impact of long-term burden of body mass index and blood pressure from childhood on adult left ventricular structure and function. J Am Heart Assoc.

[40] Gluckman PD, Seng CY, Fukuoka H, Beedle AS, Hanson MA (2007). Low birthweight and subsequent obesity in Japan. Lancet.

[41] Harder T, Schellong K, Stupin J, Dudenhausen JW, Plagemann A (2007). Where is the evidence that low birthweight leads to obesity?. Lancet.

[42] Saenger P, Czernichow P, Hughes I, Reiter EO (2007). Small for gestational age: short stature and beyond. Endocr Rev.

[43] Dumeige L, Nehlich M, Viengchareun S, Perrot J, Pussard E, Lombès M (2020). Preterm birth is associated with epigenetic programming of transgenerational hypertension in mice. Exp Mol Med.

[44] Sutherland MR, Gubhaju L, Black MJ (2011). Stereological assessment of renal development in a baboon model of preterm birth. Am J Nephrol.

[45] Wang J, Wu Y, Du B, Li Z, Ye Y, Wang H (2021). Growth patterns in early childhood and cardiovascular structure and function at 4 years old: A prospective cohort study. Nutr Metab Cardiovasc Dis.

[46] Tang A, Slopen N, Nelson CA, Zeanah CH, Georgieff MK, Fox NA (2018). Catch-up growth, metabolic, and cardiovascular risk in post-institutionalized Romanian adolescents. Pediatr Res.

[47] Jain V, Singhal A (2012). Catch up growth in low birth weight infants: striking a healthy balance. Rev Endocr Metab Disord.

[48] Ong KK (2007). Catch-up growth in small for gestational age babies: good or bad?. Curr Opin Endocrinol Diabetes Obes.

[49] Toemen L, Gaillard R, Roest AA, van der Geest RJ, Steegers EA, van der Lugt A (2020). Fetal and infant growth patterns and left and right ventricular measures in childhood assessed by cardiac MRI. Eur J Prev Cardiol.

[50] Zhang T, Li S, Bazzano L, He J, Whelton P, Chen W (2018). Trajectories of childhood blood pressure and adult left ventricular hypertrophy: the Bogalusa Heart Study. Hypertension.

[51] Harris SL, Bray H, Troughton R, Elliott J, Frampton C, Horwood J (2020). Cardiovascular outcomes in young adulthood in a population-based very low birth weight cohort. J Pediatr.

[52] Spalding KL, Arner E, Westermark PO, Bernard S, Buchholz BA, Bergmann O (2008). Dynamics of fat cell turnover in humans. Nature.

[53] Vollmer J, Casares F, Iber D. Growth and size control during development. Open Biol. 2017;7(11):170190. 10.1098/rsob.170190 PMC5717347 29142108

[54] Kapral N, Miller SE, Scharf RJ, Gurka MJ, DeBoer MD (2018). Associations between birthweight and overweight and obesity in school-age children. Pediatr Obes.

[55] Dhuper S, Abdullah RA, Weichbrod L, Mahdi E, Cohen HW (2011). Association of obesity and hypertension with left ventricular geometry and function in children and adolescents. Obesity (Silver Spring).

[56] Cuspidi C, Rescaldani M, Sala C, Grassi G (2014). Left-ventricular hypertrophy and obesity: a systematic review and meta-analysis of echocardiographic studies. J Hypertens.

[57] Mangner N, Scheuermann K, Winzer E, Wagner I, Hoellriegel R, Sandri M (2014). Childhood obesity: impact on cardiac geometry and function. JACC Cardiovasc Imaging.

[58] Erbs S, Broniecki H, Scheuermann K, Winzer E, Adam J, Spielau U (2018). Impact of weight reduction during adolescence on parameters of cardiac geometry and function in obese children. JACC Cardiovasc Imaging.

[59] Wu Y, Li Z, Du B, Ye Y, Wang H, Niu Y (2022). Different associations of systolic blood pressure and body mass index with cardiac structure and function in young children. Hypertension.

