# Supplementary material for: The association of gestational age and birthweight with blood pressure, cardiac structure, and function in 4 years old: a prospective birth cohort study
Source: BMC Med. 2023 Mar 20;21:103. doi: 10.1186/s12916-023-02812-y (PMC10029264; doi:10.1186/s12916-023-02812-y)
Supplement: Supplementary file 1 — Additional file 1: Figure S1. The flowchart of the study. Figure S2. The restricted cubic spline of BW and GA with cardiovascular parameters. Table S1. The influence of SBP and DBP on LV structure and function. Table S2-S3. The BP, LV structure and function in different BW and GA groups in linear regression models. Table S4. The association of BW and GA with BP, LV structure and function after adjusted for children weight gain from birth and BP. Table S5. The effect of BW and GA on BP, LV structure and function by putting them in the same models. Table S6. The association of BW and GA with BP, LV structure and function in fixed GA or BW groups. Table S7-S8. The effect of BW and GA on BP, LV structure and function after multiple imputation. [file 12916_2023_2812_MOESM1_ESM.docx]

**Supplemental material：**

Figure S1. The flowchart of the study.

Figure S2. The restricted cubic spline of BW (A) and GA (B) with cardiovascular parameters (n=943).

Table S1. The influence of SBP and DBP on LV structure and function.

Table S2. The BP, LV structure and function in different BW groups in linear regression models.

Table S3. The BP, LV structure and function in GA tertile groups in linear regression models.

Table S4. The association of BW and GA with BP, LV structure and function after adjusted for children weight gain from birth and BP.

Table S5. The effect of BW and GA on BP, LV structure and function by putting them in the same models.

Table S6. The association of BW and GA with BP, LV structure and function in fixed GA or BW groups.

Table S7. The independent and interaction effect of BW and GA on BP, LV structure and function after multiple imputation.

Table S8. The cardiovascular risk with GA and BW after multiple imputation.


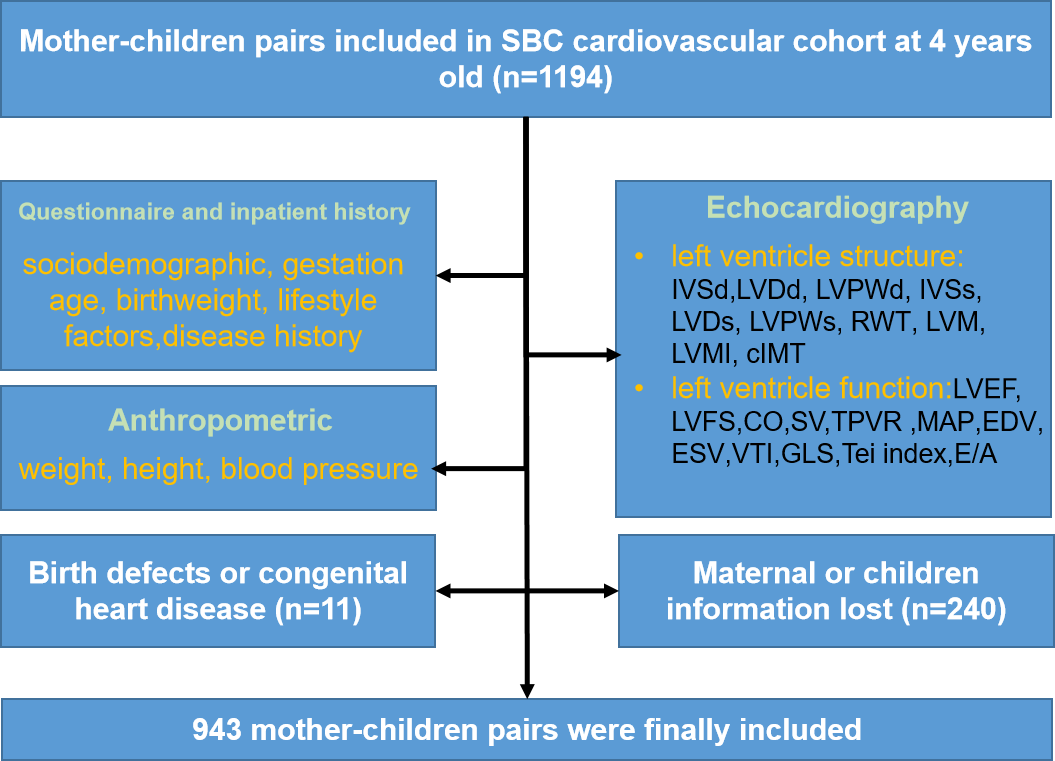


Figure S1. The flowchart of the study.


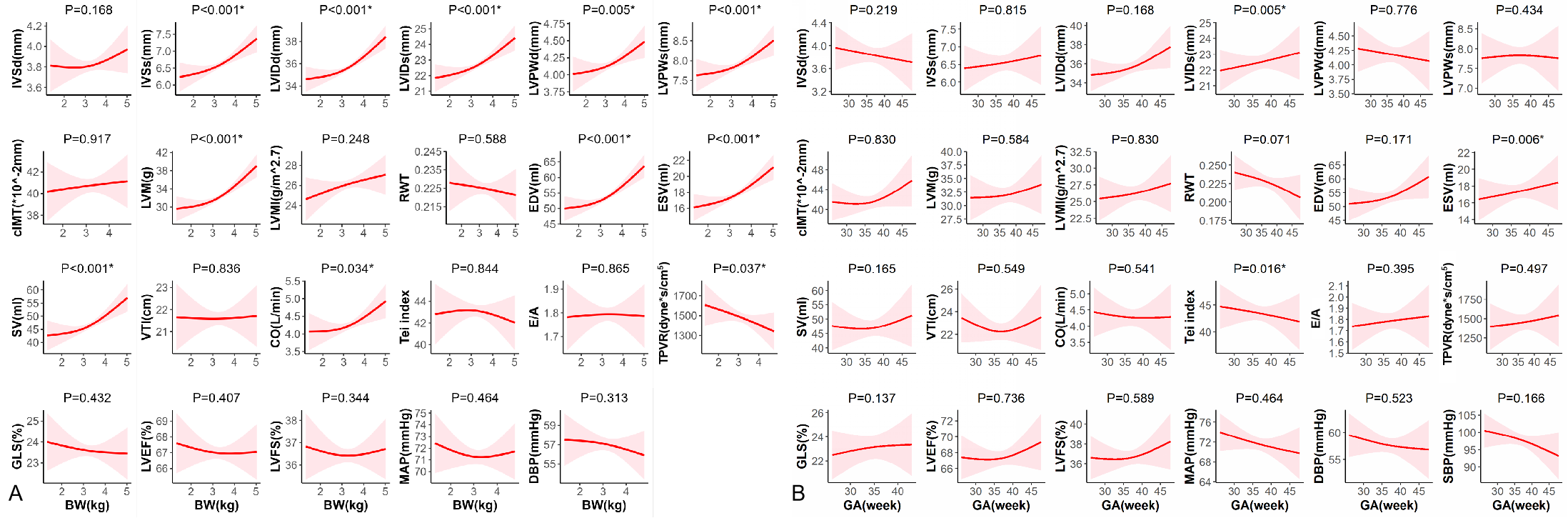


Figure S2. The restricted cubic spline of BW (A) and GA (B) with cardiovascular parameters (n=943).

The red lines indicated the predicted cardiovascular parameters derived from restricted cubic spline regression model with 3 knots at the 5th, 50th, and 95th percentiles of BW and GA. The shadow indicated the 95%CIs. Tests for non-linearity were conducted by using likelihood ratio tests.

*indicates the non-linear P value<0.05.

BW: birthweight, GA: gestational age, DBP: diastolic blood pressure, LV: left ventricle, IVSd: ventricle interventricular septal thickness in diastole, LVIDd: LV internal diameter in diastole, LVPWd: LV posterior wall thickness in diastole, IVSs: ventricle interventricular septal thickness in systole, LVIDs: LV internal diameter in systole, LVPWs: LV posterior wall thickness in systole, RWT: relative wall thickness, LVM: left ventricle mass, LVMI: LV mass index, cIMT: carotid artery intima-media thickness, LVEF: LV ejection fraction, LVFS:LV fractional shorting, CO: cardiac output, SV: stroke volume, TPVR: total peripheral vascular resistance , MAP: mean arterial pressure, EDV: end diastolic volume, ESV: end systolic volume, VTI: velocity time integral, GLS: global longitudinal strain.

Table S1. The influence of SBP and DBP on LV structure and function.

|  | SBP | | DBP | |
| --- | --- | --- | --- | --- |
|  | Unadjusted | Adjusted | Unadjusted | Adjusted |
| **LV structure** |  |  |  |  |
| IVSd (mm) | 0.00(0.00,0.01) | 0.00(0.00,0.01) | 0.00(-0.01,0.00) | 0.00(-0.01,0.00) |
| LVIDd (mm) | **0.06(0.04,0.08)** | **0.04(0.02,0.06)** | -0.02(-0.05,0.01) | -0.01(-0.04,0.01) |
| LVPWd (mm) | **0.01(0.01,0.02)** | **0.01(0.00,0.01)** | 0.00(-0.01,0.01) | 0.00(-0.01,0.01) |
| IVSs (mm) | **0.02(0.02,0.03)** | **0.02(0.01,0.03)** | 0.01(0.00,0.02) | 0.01(0.00,0.02) |
| LVIDs (mm) | **0.02(0.01,0.04)** | 0.01(0.00,0.03) | -0.02(-0.04,0.00) | -0.02(-0.04,0.00) |
| LVPWs (mm) | **0.02(0.01,0.03)** | **0.02(0.01,0.03)** | 0.01(0.00,0.02) | 0.01(0.00,0.02) |
| RWT | 0.00(0.00,0.00) | 0.00(0.00,0.00) | 0.00(0.00,0.00) | 0.00(0.00,0.00) |
| LVM (g) | **0.15(0.10,0.20)** | **0.11(0.06,0.16)** | -0.03(-0.10,0.03) | -0.02(-0.08,0.04) |
| LVMI (g/cm^2.7^) | 0.02(-0.02,0.06) | 0.00(-0.05,0.04) | **-0.07(-0.12,-0.02)** | **-0.06(-0.11,-0.01)** |
| cIMT (*10^-2^mm) | 0.01(-0.04,0.06) | 0.01(-0.04,0.05) | 0.00(-0.06,0.06) | 0.01(-0.05,0.06) |
| **LV function** |  |  |  |  |
| LVEF (%) | **0.04(0.01,0.08)** | **0.04(0.01,0.08)** | 0.03(-0.01,0.07) | 0.03(-0.01,0.07) |
| LVFS (%) | **0.04(0.01,0.07)** | **0.04(0.01,0.07)** | 0.02(-0.01,0.05) | 0.02(-0.01,0.05) |
| Tei index (%) | 0.00(-0.05,0.05) | 0.00(-0.05,0.05) | **0.13(0.07,0.19)** | **0.13(0.07,0.19)** |
| E/A | **-0.01(-0.01,0.00)** | **-0.01(-0.01,0.00)** | **-0.01(-0.01,0.00)** | **-0.01(-0.01,0.00)** |
| CO (L/min) | **0.02(0.01,0.03)** | **0.02(0.01,0.03)** | **0.01(0.00,0.02)** | **0.01(0.00,0.02)** |
| SV (ml) | **0.16(0.06,0.26)** | 0.09(-0.01,0.19) | -0.06(-0.18,0.07) | -0.03(-0.15,0.09) |
| TPVR (dyne*s/cm^5^) | **4.65(1.10,8.21)** | **7.01(3.44,10.59)** | **13.78(9.47,18.08)** | **13.07(8.81,17.34)** |
| EDV (ml) | **0.20(0.13,0.27)** | **0.15(0.08,0.22)** | -0.07(-0.16,0.02) | -0.04(-0.13,0.05) |
| ESV (ml) | **0.04(0.01,0.08)** | 0.03(0.00,0.06) | **-**0.04(-0.08,0.00) | -0.03(-0.07,0.00) |
| VTI (cm) | **0.04(0.02,0.07)** | **0.04(0.01,0.07)** | **-0.04(-0.07,-0.01)** | **-0.04(-0.07,-0.01)** |
| GLS (%) | **-0.04(-0.06,-0.01)** | **-0.03(-0.06,-0.01)** | **-0.04(-0.08,-0.01)** | **-0.04(-0.07,-0.01)** |

The data were presented as β(95%CI) in linear regression models. The missing values were not inputted (Unadjusted: n=943, Adjusted: n=786).

The bold values were P<0.05.

Adjusted for maternal nationality, scholarship, income, HDP, GDM, drink history, passive smoke history, gender of children.

SBP: systolic blood pressure, DBP: diastolic blood pressure, LV: left ventricle, IVSd: ventricle interventricular septal thickness in diastole, LVIDd: LV internal diameter in diastole, LVPWd: LV posterior wall thickness in diastole, IVSs: ventricle interventricular septal thickness in systole, LVIDs: LV internal diameter in systole, LVPWs: LV posterior wall thickness in systole, RWT: relative wall thickness, LVM: left ventricle mass, LVMI: LV mass index, cIMT: carotid artery intima-media thickness, LVEF: LV ejection fraction, LVFS:LV fractional shorting, CO: cardiac output, SV: stroke volume, TPVR: total peripheral vascular resistance , MAP: mean arterial pressure, EDV: end diastolic volume, ESV: end systolic volume, VTI: velocity time integral, GLS: global longitudinal strain, HDP: hypertensive disorders in pregnancy, GDM: gestational diabetes mellitus.

Table S2. The BP, LV structure and function in different BW groups in linear regression models.

|  | Crude | | Model 1 | | Model 2 | |
| --- | --- | --- | --- | --- | --- | --- |
|  | LBW | MBW | LBW | MBW | LBW | MBW |
| **Blood pressure** | |  |  |  |  |  |
| SBP(mmHg) | -1.02(-2.85,0.80) | 1.08(-0.61,2.75) | -2.30(-4.84,0.23) | -0.50(-2.64,1.64) | -2.04(-4.51,0.42) | -1.77(-3.86,0.33) |
| DBP(mmHg) | 0.02(-1.45,1.48) | -0.88(-2.23,0.47) | -1.07(-3.25,1.12) | -1.59(-3.44,0.25) | -1.11(-3.31,1.09) | **-2.08(-3.95,-0.21)** |
| MAP(mmHg) | -0.24(-1.64,1.17) | -0.34(-1.64,0.95) | -1.17(-3.23,0.90) | -0.99(-2.74,0.76) | -1.15(-3.21,0.91) | -1.67(-3.43,0.09) |
| **LV structure** | |  |  |  |  |  |
| IVSd(mm) | -0.08(-0.21,0.05) | 0.03(-0.09,0.15) | -0.11(-0.28,0.06) | -0.01(-0.16,0.14) | -0.11(-0.28,0.06) | -0.02(-0.17,0.14) |
| LVIDd(mm) | **-0.63(-1.23,-0.03)** | **0.68(0.13,1.22)** | -0.41(-1.20,0.38) | **1.22(0.52,1.92)** | -0.34(-1.11,0.42) | **0.89(0.20,1.58)** |
| LVPWd(mm) | **-0.14(-0.28,0.00)** | 0.12(-0.01,0.24) | -0.16(-0.35,0.03) | 0.06(-0.11,0.22) | -0.15(-0.34,0.03) | 0.02(-0.15,0.18) |
| IVSs(mm) | **-0.24(-0.47,-0.01)** | 0.18(-0.03,0.39) | -0.19(-0.49,0.12) | 0.19(-0.08,0.46) | -0.17(-0.47,0.13) | 0.10(-0.17,0.37) |
| LVIDs(mm) | -0.44(-0.90,0.03) | **0.43(0.00,0.85)** | -0.50(-1.13,0.13) | **0.93(0.37,1.49)** | -0.46(-1.08,0.16) | **0.71(0.16,1.27)** |
| LVPWs(mm) | -0.12(-0.34,0.10) | **0.24(0.04,0.44)** | 0.06(-0.25,0.37) | 0.21(-0.06,0.49) | 0.08(-0.21,0.38) | 0.09(-0.18,0.36) |
| RWT | 0.00(-0.01,0.01) | 0.00(-0.01,0.01) | -0.01(-0.02,0.01) | -0.01(-0.02,0.01) | -0.01(-0.02,0.01) | -0.01(-0.02,0.01) |
| LVM(g) | **-2.17(-3.61,-0.73)** | **1.88(0.56,3.19)** | **-2.11(-4.06,-0.16)** | **2.42(0.68,4.16)** | **-1.96(-3.86,-0.06)** | 1.64(-0.08,3.35) |
| LVMI(g/cm^2.7^) | -0.40(-1.54,0.74) | -0.15(-1.19,0.89) | -0.02(-1.63,1.58) | -0.37(-1.80,1.05) | 0.10(-1.47,1.66) | -0.98(-2.39,0.43) |
| cIMT(*10^-2^mm) | **-1.57(-2.91,-0.23)** | -0.34(-1.68,1.00) | **-1.92(-3.79,0.05)** | -1.03(-2.81,0.76) | **-1.95(-3.84,-0.07)** | -0.98(-2.79,0.84) |
| **LV function** | |  |  |  |  |  |
| LVEF(%) | 0.11(-0.89,1.10) | 0.09(-0.82,1.00) | 0.80(-0.58,2.19) | -0.41(-1.64,0.82) | 0.80(-0.59,2.19) | -0.39(-1.64,0.86) |
| LVFS(%) | 0.06(-0.73,0.84) | 0.15(-0.57,0.86) | 0.64(-0.44,1.73) | -0.22(-1.18,0.75) | 0.64(-0.44,1.73) | -0.21(-1.19,0.77) |
| Tei index(%) | 0.52(-0.95,1.99) | -0.95(-2.32,0.43) | 1.23(-0.84,3.29) | -0.61(-2.49,1.26) | 1.20(-0.87,3.27) | -0.48(-2.38,1.42) |
| E/A | -0.04(-0.11,0.04) | 0.00(-0.07,0.07) | -0.03(-0.14,0.08) | -0.05(-0.15,0.04) | -0.03(-0.14,0.09) | -0.04(-0.14,0.05) |
| CO(L/min) | -0.07(-0.35,0.21) | 0.20(-0.06,0.47) | -0.08(-0.46,0.30) | 0.34(-0.01,0.70) | -0.05(-0.42,0.32) | 0.22(-0.13,0.57) |
| SV(ml) | -2.31(-5.27,0.66) | **4.06(1.23,6.89)** | -2.73(-6.76,1.31) | **6.69(2.95,10.42)** | -2.42(-6.39,1.54) | **5.36(1.65,9.08)** |
| TPVR(dyne*s/cm^5^) | 79.02(-32.12,190.17) | -0.24(-1.64,1.17) | 107.20(-54.68,269.08) | -96.01(-239.37,47.35) | 101.30(-59.96,262.56) | -70.54(-214.81,73.73) |
| EDV(ml) | **-2.31(-4.43,-0.18)** | **2.46(0.52,4.41)** | -1.58(-4.40,1.25) | **4.51(2.00,7.03)** | -1.34(-4.09,1.40) | **3.31(0.84,5.78)** |
| ESV(ml) | -0.89(-1.79,0.01) | **0.90(0.08,1.72)** | -0.98(-2.21,0.25) | **1.87(0.78,2.96)** | -0.90(-2.10,0.31) | **1.44(0.36,2.52)** |
| VTI(cm) | -0.03(-0.81,0.75) | -0.13(-0.87,0.62) | -0.15(-1.23,0.94) | 0.20(-0.80,1.20) | -0.09(-1.16,0.99) | -0.05(-1.06,0.96) |
| GLS(%) | 0.29(-0.36,0.95) | 0.18(-0.53,0.89) | 0.44(-0.50,1.39) | -0.24(-1.27,0.79) | 0.44(-0.51,1.38) | -0.08(-1.12,0.96) |

The data were presented as β(95%CI) in linear regression models.The missing values were not inputted (Crude model: n=943, Model 1: n=786, Model 2: n=583).

The bold values were P<0.05.

Model 1: adjusted for maternal nationality, scholarship, income, HDP, GDM, drink history, passive smoke history and gender of children.

Model 2: Model 1 + BMI at 4 years old.

NBW as reference.

LBW: low birthweight, NBW: normal birthweight, MBW: macrosomia, SBP: systolic blood pressure, DBP: diastolic blood pressure, LV: left ventricle, IVSd: ventricle interventricular septal thickness in diastole, LVIDd: LV internal diameter in diastole, LVPWd: LV posterior wall thickness in diastole, IVSs: ventricle interventricular septal thickness in systole, LVIDs: LV internal diameter in systole, LVPWs: LV posterior wall thickness in systole, RWT: relative wall thickness, LVM: left ventricle mass, LVMI: LV mass index, cIMT: carotid artery intima-media thickness, LVEF: LV ejection fraction, LVFS:LV fractional shorting, CO: cardiac output, SV: stroke volume, TPVR: total peripheral vascular resistance , MAP: mean arterial pressure, EDV: end diastolic volume, ESV: end systolic volume, VTI: velocity time integral, GLS: global longitudinal strain, BMI: body mass index, HDP: hypertensive disorders in pregnancy, GDM: gestational diabetes mellitus.

Table S3. The BP, LV structure and function in GA tertile groups in linear regression models.

|  | Crude | | Model 1 | | Model 2 | |
| --- | --- | --- | --- | --- | --- | --- |
|  | GA Tertile 1 | GA Tertile 3 | GA Tertile 1 | GA Tertile 3 | GA Tertile 1 | GA Tertile 3 |
| **Blood pressure** | |  |  |  |  |  |
| SBP(mmHg) | 0.05(-1.07,1.17) | -0.91(-2.06,0.24) | -0.24(-1.65,1.17) | **-1.58(-3.00,-0.16)** | -0.19(-1.55,1.18) | **-1.38(-2.75,-0.02)** |
| DBP(mmHg) | 0.11(-0.80,1.02) | -0.63(-1.56,0.31) | -0.32(-1.56,0.91) | -0.45(-1.68,0.79) | -0.35(-1.58,0.88) | -0.51(-1.74,0.73) |
| MAP(mmHg) | 0.12(-0.74,0.98) | -0.64(-1.52,0.24) | -0.09(-1.23,1.06) | -1.10(-2.26,0.06) | -0.08(-1.22,1.05) | -1.10(-2.25,0.04) |
| **LV structure** | |  |  |  |  |  |
| IVSd(mm) | 0.01(-0.08,0.09) | -0.02(-0.10,0.07) | 0.03(-0.07,0.13) | -0.03(-0.13,0.07) | 0.03(-0.07,0.13) | -0.03(-0.13,0.07) |
| LVIDd(mm) | -0.26(-0.64,0.11) | 0.20(-0.18,0.58) | -0.43(-0.89,0.03) | 0.02(-0.44,0.47) | -0.41(-0.85,0.03) | 0.02(-0.42,0.46) |
| LVPWd(mm) | -0.01(-0.10,0.08) | 0.01(-0.08,0.10) | -0.02(-0.13,0.09) | -0.04(-0.15,0.07) | -0.01(-0.12,0.09) | -0.04(-0.15,0.07) |
| IVSs(mm) | -0.10(-0.24,0.05) | -0.01(-0.15,0.14) | -0.07(-0.24,0.11) | -0.06(-0.24,0.11) | -0.06(-0.24,0.11) | -0.06(-0.23,0.11) |
| LVIDs(mm) | -0.13(-0.42,0.16) | -0.02(-0.32,0.27) | -0.36(-0.73,0.01) | -0.06(-0.42,0.31) | -0.35(-0.70,0.01) | -0.05(-0.41,0.31) |
| LVPWs(mm) | -0.06(-0.20,0.08) | 0.00(-0.14,0.14) | -0.07(-0.25,0.11) | -0.02(-0.20,0.16) | -0.07(-0.24,0.11) | -0.02(-0.19,0.16) |
| RWT | 0.00(0.00,0.01) | 0.00(-0.01,0.00) | 0.00(-0.01,0.01) | 0.00(-0.01,0.00) | 0.00(-0.01,0.01) | 0.00(-0.01,0.00) |
| LVM(g) | -0.60(-1.52,0.32) | 0.16(-0.77,1.09) | -0.83(-1.97,0.32) | -0.48(-1.62,0.67) | -0.78(-1.88,0.33) | -0.46(-1.57,0.64) |
| LVMI(g/cm^2.7^) | -0.22(-0.94,0.51) | 0.18(-0.55,0.92) | -0.12(-1.05,0.81) | 0.09(-0.84,1.02) | -0.09(-0.99,0.82) | 0.10(-0.81,1.01) |
| cIMT(*10^-2^mm) | 0.29(-0.55,1.12) | **0.93(0.07,1.79)** | 0.07(-1.02,1.16) | 0.93(-0.15,2.01) | 0.07(-1.02,1.16) | 0.93(-0.16,2.01) |
| **LV function** | |  |  |  |  |  |
| LVEF(%) | -0.04(-0.64,0.56) | 0.57(-0.05,1.18) | 0.44(-0.36,1.23) | 0.28(-0.52,1.08) | 0.44(-0.36,1.23) | 0.28(-0.52,1.08) |
| LVFS(%) | -0.10(-0.57,0.38) | 0.41(-0.08,0.89) | 0.29(-0.33,0.91) | 0.21(-0.41,0.83) | 0.29(-0.33,0.92) | 0.21(-0.41,0.84) |
| Tei index(%) | 0.71(-0.21,1.63) | -0.12(-1.06,0.82) | 0.81(-0.38,2.00) | 0.58(-0.61,1.77) | 0.82(-0.37,2.02) | 0.59(-0.60,1.78) |
| E/A | 0.00(-0.05,0.04) | 0.01(-0.04,0.06) | -0.01(-0.08,0.05) | 0.01(-0.05,0.07) | -50.02(-0.08,0.05) | 0.01(-0.05,0.08) |
| CO(L/min) | -0.02(-0.19,0.15) | -0.01(-0.18,0.16) | -0.04(-0.26,0.18) | -0.06(-0.28,0.15) | -0.04(-0.26,0.18) | -0.06(-0.27,0.16) |
| SV(ml) | -0.48(-2.32,1.36) | 0.30(-1.58,2.17) | -1.06(-3.44,1.33) | -0.40(-2.76,1.96) | -1.03(-3.37,1.30) | -0.37(-2.68,1.94) |
| TPVR(dyne*s/cm^5^) | 4.02(-63.75,71.79) | 0.09(-68.76,68.93) | 17.16(-75.42,109.74) | 6.50(-85.21,98.21) | 17.86(-74.14,109.86) | 6.15(-84.98,97.28) |
| EDV(ml) | -0.99(-2.32,0.34) | 0.61(-0.74,1.96) | -1.64(-3.28,0.01) | -0.01(-1.65,1.63) | -1.56(-3.14,0.02) | 0.02(-1.56,1.59) |
| ESV(ml) | -0.26(-0.81,0.30) | -0.07(-0.63,0.49) | -0.71(-1.43,0.00) | -0.12(-0.84,0.59) | -0.69(-1.39,0.01) | -0.12(-0.81,0.58) |
| VTI(cm) | 0.12(-0.36,0.61) | 0.15(-0.34,0.64) | 0.15(-0.48,0.78) | 0.09(-0.54,0.72) | 0.15(-0.48,0.78) | 0.09(-0.53,0.71) |
| GLS(%) | 0.03(-0.42,0.48) | 0.01(-0.44,0.46) | 0.14(-0.45,0.74) | -0.28(-0.86,0.30) | 0.14(-0.45,0.73) | -0.26(-0.84,0.31) |

The data were presented as β(95%CI) in linear regression models. The missing values were not inputted (Crude model: n=943, Model 1: n=786, Model 2: n=583).

The bold values were P<0.05. The GA was divided into 3 tertile groups.

Model 1: adjusted for maternal nationality, scholarship, income, HDP, GDM, drink history, passive smoke history and gender of children.

Model 2: Model 1 + BMI at 4 years old.

GA was grouped by tertiles (Tertile 1:GA≤38.85 weeks, Tertile 2: GA 38.85-39.85 weeks, Tertile 3:GA≥39.85 weeks). Tertile 2 as reference.

GA: gestational age, SBP: systolic blood pressure, DBP: diastolic blood pressure, LV: left ventricle, IVSd: ventricle interventricular septal thickness in diastole, LVIDd: LV internal diameter in diastole, LVPWd: LV posterior wall thickness in diastole, IVSs: ventricle interventricular septal thickness in systole, LVIDs: LV internal diameter in systole, LVPWs: LV posterior wall thickness in systole, RWT: relative wall thickness, LVM: left ventricle mass, LVMI: LV mass index, cIMT: carotid artery intima-media thickness, LVEF: LV ejection fraction, LVFS:LV fractional shorting, CO: cardiac output, SV: stroke volume, TPVR: total peripheral vascular resistance , MAP: mean arterial pressure, EDV: end diastolic volume, ESV: end systolic volume, VTI: velocity time integral, GLS: global longitudinal strain, BMI: body mass index, HDP: hypertensive disorders in pregnancy, GDM: gestational diabetes mellitus.

Table S4. The association of BW and GA with BP, LV structure and function after adjusted for children weight gain from birth and BP.

|  | BW | | GA | |
| --- | --- | --- | --- | --- |
| **Blood pressure** | Model 1 | Model 2 | Model 1 | Model 2 |
| SBP(mmHg) | 0.02(-1.51,1.55) | / | -0.20(-0.66,0.26) | / |
| DBP(mmHg) | -0.51(-1.90,0.88) | / | 0.04(-0.38,0.46) | / |
| MAP(mmHg) | -0.33(-1.62,0.96) | / | -0.20(-0.58,0.19) | / |
| **LV structure** |  |  |  |  |
| IVSd(mm) | 0.08(-0.03,0.19) | 0.07(-0.05,0.19) | -0.03(-0.06,0.01) | -0.03(-0.06,0.01) |
| LVIDd(mm) | **0.60(0.13,1.07)** | **0.59(0.10,1.08)** | 0.12(-0.02,0.27) | 0.09(-0.06,0.24) |
| LVPWd(mm) | **0.14(0.02,0.26)** | **0.13(0.00,0.26)** | -0.03(-0.06,0.01) | -0.02(-0.06,0.01) |
| IVSs(mm) | **0.23(0.03,0.42)** | **0.21(0.00,0.41)** | -0.02(-0.08,0.04) | -0.01(-0.07,0.05) |
| LVIDs(mm) | **0.68(0.29,1.06)** | **0.64(0.24,1.03)** | 0.08(-0.03,0.20) | 0.06(-0.06,0.18) |
| LVPWs(mm) | 0.07(-0.12,0.27) | 0.09(-0.11,0.30) | -0.01(-0.07,0.05) | -0.02(-0.08,0.04) |
| RWT | 0.00(0.00,0.01) | 0.00(0.00,0.01) | 0.00(0.00,0.00) | 0.00(0.00,0.00) |
| LVM(g) | **2.11(0.93,3.29)** | **2.01(0.76,3.26)** | -0.06(-0.42,0.30) | -0.10(-0.48,0.28) |
| LVMI(g/cm^2.7^) | 0.50(-0.56,1.55) | 0.49(-0.63,1.61) | 0.06(-0.26,0.38) | -0.01(-0.35,0.34) |
| cIMT(*10-2mm) | -0.18(-1.47,1.11) | -0.40(-1.71,0.92) | 0.12(-0.28,0.51) | 0.19(-0.22,0.59) |
| **LV function** |  |  |  |  |
| LVEF(%) | -0.89(-1.80,0.01) | -0.78(-1.72,0.16) | -0.08(-0.35,0.20) | -0.07(-0.36,0.21) |
| LVFS(%) | -0.64(-1.35,0.08) | -0.54(-1.28,0.20) | -0.05(-0.27,0.16) | -0.05(-0.28,0.17) |
| Tei index(%) | 0.35(-1.03,1.72) | 0.35(-1.04,1.75) | -0.11(-0.52,0.30) | -0.06(-0.48,0.36) |
| E/A | -0.03(-0.10,0.04) | -0.03(-0.10,0.05) | 0.02(0.00,0.04) | 0.02(0.00,0.04) |
| CO(L/min) | **0.27(0.02,0.52)** | **0.39(0.14,0.63)** | -0.05(-0.12,0.03) | -0.05(-0.12,0.03) |
| SV(ml) | **4.77(2.15,7.40)** | **5.59(2.89,8.29)** | -0.57(-1.35,0.22) | -0.69(-1.50,0.12) |
| TPVR(dyne*s/cm^5^) | **-117.91(-222.90,-12.93)** | **-142.90(-245.08,-40.73)** | 16.89(-14.66,48.45) | 22.85(-7.91,53.61) |
| EDV(ml) | **2.22(0.54,3.90)** | **2.23(0.48,3.98)** | 0.45(-0.06,0.96) | 0.35(-0.18,0.89) |
| ESV(ml) | **1.28(0.54,2.02)** | **1.22(0.45,1.99)** | 0.17(-0.05,0.40) | 0.14(-0.09,0.37) |
| VTI(cm) | 0.02(-0.70,0.75) | 0.12(-0.63,0.86) | -0.03(-0.25,0.19) | -0.04(-0.27,0.18) |
| GLS(%) | -0.34(-1.00,0.32) | -0.29(-0.99,0.41) | 0.02(-0.18,0.21) | -0.02(-0.22,0.18) |

The data were presented as β(95%CI) in linear regression models.The BW and GA were analyzed together in the same linear regression models.

Model 1 (n=583): Adjusted for maternal nationality, scholarship, income, HDP, GDM, drink history, passive smoke history, gender and weight gain from birth to 4 of children.

Model 2 (n=583): Model 1 + SBP of children.

The bold values were P<0.05.

BW: birthweight, GA: gestational age, SBP: systolic blood pressure, DBP: diastolic blood pressure, LV: left ventricle, IVSd: ventricle interventricular septal thickness in diastole, LVIDd: LV internal diameter in diastole, LVPWd: LV posterior wall thickness in diastole, IVSs: ventricle interventricular septal thickness in systole, LVIDs: LV internal diameter in systole, LVPWs: LV posterior wall thickness in systole, RWT: relative wall thickness, LVM: left ventricle mass, LVMI: LV mass index, cIMT: carotid artery intima-media thickness, LVEF: LV ejection fraction, LVFS:LV fractional shorting, CO: cardiac output, SV: stroke volume, TPVR: total peripheral vascular resistance , MAP: mean arterial pressure, EDV: end diastolic volume, ESV: end systolic volume, VTI: velocity time integral, GLS: global longitudinal strain, HDP: hypertensive disorders in pregnancy, GDM: gestational diabetes mellitus.

Table S5. The effect of BW and GA on BP, LV structure and function by putting them in the same models.

|  | BW | | | | | | GA | | | | | | |
| --- | --- | --- | --- | --- | --- | --- | --- | --- | --- | --- | --- | --- | --- |
|  | β | Standardized β | P | Adjusted β^a^ | Standardized adjusted β | P | β | Standardized β | P | | Adjusted β^a^ | Standardized adjusted β | P |
| **Blood pressure** | |  |  |  |  |  |  |  |  | |  |  |  |
| SBP(mmHg) | **2.08** | **0.13** | **<0.001** | 1.28 | 0.08 | 0.11 | **-0.74** | **-0.14** | | **<0.001** | **-0.66** | **-0.14** | **0.005** |
| DBP(mmHg) | -0.34 | -0.03 | 0.50 | 0.01 | 0.01 | 0.99 | -0.07 | -0.02 | | 0.65 | -0.15 | -0.04 | 0.47 |
| MAP(mmHg) | 0.34 | 0.03 | 0.48 | 0.38 | 0.03 | 0.56 | **-0.31** | **-0.08** | | **0.04** | **-0.44** | **-0.12** | **0.02** |
| **LV structure** |  |  |  |  |  |  |  |  | |  |  |  |  |
| IVSd(mm) | 0.08 | 0.07 | 0.09 | 0.09 | 0.09 | 0.10 | -0.02 | -0.06 | | 0.16 | -0.03 | -0.10 | 0.05 |
| LVIDd(mm) | **1.23** | **0.23** | **<0.001** | **1.15** | **0.22** | **<0.001** | -0.11 | -0.07 | | 0.08 | -0.07 | -0.04 | 0.36 |
| LVPWd(mm) | **0.17** | **0.14** | **<0.001** | **0.19** | **0.16** | **0.002** | -0.02 | -0.06 | | 0.10 | **-0.04** | **-0.11** | **0.02** |
| IVSs(mm) | **0.39** | **0.19** | **<0.001** | **0.33** | **0.16** | **<0.001** | **-0.05** | **-0.08** | | **0.05** | -0.05 | -0.07 | 0.13 |
| LVIDs(mm) | **0.86** | **0.21** | **<0.001** | **1.04** | **0.25** | **<0.001** | -0.09 | -0.07 | | 0.07 | -0.05 | -0.04 | 0.38 |
| LVPWs(mm) | **0.34** | **0.17** | **<0.001** | **0.20** | **0.10** | **0.04** | **-0.06** | **-0.10** | | **0.01** | -0.04 | -0.07 | 0.15 |
| RWT | 0.00 | 0.02 | 0.66 | 0.00 | 0.04 | 0.41 | 0.00 | -0.03 | | 0.51 | 0.00 | -0.08 | 0.12 |
| LVM(g) | **3.24** | **0.25** | **<0.001** | **3.34** | **0.26** | **<0.001** | **-0.40** | **-0.10** | | **0.01** | **-0.47** | **-0.12** | **0.01** |
| LVMI(g/cm^2.7^) | 0.49 | 0.05 | 0.20 | 0.42 | 0.04 | 0.43 | 0.10 | 0.03 | | 0.42 | 0.09 | 0.03 | 0.57 |
| cIMT(*10^-2^mm) | 0.37 | 0.04 | 0.45 | -0.11 | -0.01 | 0.87 | -0.06 | -0.02 | | 0.70 | 0.08 | 0.03 | 0.68 |
| **LV function** | |  |  |  |  |  |  |  | |  |  |  |  |
| LVEF(%) | -0.23 | -0.03 | 0.49 | **-0.97** | **-0.11** | **0.03** | 0.05 | 0.02 | | 0.68 | -0.02 | -0.01 | 0.90 |
| LVFS(%) | -0.07 | -0.01 | 0.81 | -0.67 | -0.10 | 0.06 | 0.02 | 0.01 | | 0.83 | -0.02 | -0.01 | 0.87 |
| Tei index(%) | 0.19 | 0.01 | 0.71 | 0.19 | 0.01 | 0.78 | -0.24 | -0.06 | | 0.15 | -0.08 | -0.02 | 0.69 |
| E/A | -0.01 | -0.01 | 0.79 | -0.03 | -0.04 | 0.48 | 0.01 | 0.02 | | 0.52 | 0.02 | 0.07 | 0.13 |
| CO(L/min) | **0.36** | **0.15** | **<0.001** | **0.42** | **0.17** | **0.001** | **-0.07** | **-0.09** | | **0.02** | **-0.09** | **-0.12** | **0.02** |
| SV(ml) | **5.61** | **0.21** | **<0.001** | **6.59** | **0.24** | **<0.001** | **-1.01** | **-0.12** | | **0.002** | **-1.14** | **-0.14** | **0.004** |
| TPVR(dyne*s/cm^5^) | **-120.58** | **-0.12** | **0.002** | **-149.78** | **-0.15** | **0.005** | **25.45** | **0.08** | | **0.04** | 28.75 | 0.10 | 0.06 |
| EDV(ml) | **4.44** | **0.24** | **<0.001** | **4.20** | **0.22** | **<0.001** | -0.42 | -0.07 | | 0.07 | -0.26 | -0.05 | 0.35 |
| ESV(ml) | **1.70** | **0.21** | **<0.001** | **1.99** | **0.25** | **<0.001** | -0.19 | -0.07 | | 0.05 | -0.1 | -0.04 | 0.40 |
| VTI(cm) | 0.19 | 0.03 | 0.49 | 0.34 | 0.05 | 0.35 | -0.11 | -0.05 | | 0.23 | -0.12 | -0.06 | 0.27 |
| GLS(%) | -0.28 | -0.06 | 0.24 | -0.55 | -0.11 | 0.10 | 0.09 | 0.06 | | 0.26 | 0.08 | 0.06 | 0.39 |

The BW and GA were analyzed together in the same linear regression models. The missing values were not inputted (Unadjusted: n=943, Adjusted: n=786).

a Adjusted for maternal nationality, scholarship, income, HDP, GDM, drink history, passive smoke history and gender of children.

The bold values were P<0.05.

BW: birthweight, GA: gestational age, SBP: systolic blood pressure, DBP: diastolic blood pressure, LV: left ventricle, IVSd: ventricle interventricular septal thickness in diastole, LVIDd: LV internal diameter in diastole, LVPWd: LV posterior wall thickness in diastole, IVSs: ventricle interventricular septal thickness in systole, LVIDs: LV internal diameter in systole, LVPWs: LV posterior wall thickness in systole, RWT: relative wall thickness, LVM: left ventricle mass, LVMI: LV mass index, cIMT: carotid artery intima-media thickness, LVEF: LV ejection fraction, LVFS:LV fractional shorting, CO: cardiac output, SV: stroke volume, TPVR: total peripheral vascular resistance , MAP: mean arterial pressure, EDV: end diastolic volume, ESV: end systolic volume, VTI: velocity time integral, GLS: global longitudinal strain, HDP: hypertensive disorders in pregnancy, GDM: gestational diabetes mellitus.

Table S6. The association of BW and GA with BP, LV structure and function in fixed GA or BW groups.

|  | BW | | GA | | |  |
| --- | --- | --- | --- | --- | --- | --- |
|  | Preterm | Term | LBW | NBW | MBW |  |
| **Blood pressure** | |  |  |  |  |  |
| SBP(mmHg) | **-4.22(-8.02,-0.42)** | 0.64(-0.93,2.20) | **-0.58(-1.13,-0.04)** | **-0.55(-1.04,-0.07)** | 0.72(-3.35,4.80) |  |
| DBP(mmHg) | 1.12(-2.58,4.81) | -0.25(-1.59,1.10) | -0.32(-1.49,0.85) | -0.16(-0.58,0.26) | -0.74(-4.67,3.20) |  |
| MAP(mmHg) | -0.94(-5.02,3.14) | -0.04(-1.29,1.21) | **-0.45(-0.88,-0.03)** | -0.39(-0.78,0.01) | -0.77(-4.08,2.53) |  |
| **LV structure** |  |  |  |  |  |  |
| IVSd(mm) | -0.15(-0.34,0.04) | 0.09(-0.02,0.20) | -0.06(-0.13,0.01) | -0.02(-0.05,0.02) | -0.04(-0.21,0.14) |  |
| LVIDd(mm) | -0.09(-1.54,1.36) | **1.37(0.87,1.86)** | -0.07(-0.43,0.26) | 0.11(-0.06,0.27) | 0.50(-0.65,1.65) |  |
| LVPWd(mm) | -0.03(-0.41,0.35) | **0.19(0.07,0.30)** | -0.05(-0.16,0.06) | -0.03(-0.07,0.01) | -0.13(-0.34,0.08) |  |
| IVSs(mm) | 0.01(-0.53,0.56) | **0.32(0.13,0.51)** | 0.08(-0.12,0.28) | 0.01(-0.06,0.07) | 0.30(-0.19,0.80) |  |
| LVIDs(mm) | 0.77(-0.42,1.96) | **1.19(0.80,1.58)** | -0.03(-0.35,0.33) | 0.05(-0.08,0.18) | 0.10(-0.94,1.14) |  |
| LVPWs(mm) | -0.33(-0.90,0.25) | **0.25(0.06,0.44)** | -0.06(-0.24,0.11) | 0.01(-0.05,0.08) | -0.03(-0.58,0.53) |  |
| RWT | 0.00(-0.03,0.02) | 0.00(-0.01,0.01) | 0.00(-0.01,0.00) | 0.00(-0.01,0.00) | -0.01(-0.02,0.00) |  |
| LVM(g) | -1.17(-4.19,1.86) | **3.63(2.41,4.86)** | -0.63(-1.53,0.27) | -0.07(-0.47,0.34) | -0.34(-3.29,2.61) |  |
| LVMI(g/cm^2.7^) | -0.39(-2.90,2.11) | 0.71(-0.32,1.73) | -0.27(-0.95,0.41) | 0.07(-0.26,0.41) | 0.09(-2.30,2.47) |  |
| cIMT(*10^-2^mm) | 0.06(-3.42,3.54) | 0.07(-1.22,1.37) | **1.36(0.25,2.47)** | **0.39(0.01,0.77)** | 2.04(-1.84,5.92) |  |
| **LV function** |  |  |  |  |  |  |
| LVEF(%) | **-2.91(-5.14,-0.68)** | **-1.09(-1.97,-0.21)** | -0.03(-0.91,0.85) | 0.01(-0.28,0.29) | 0.20(-1.88,2.28) |  |
| LVFS(%) | **-2.31(-4.02,-0.59)** | **-0.75(-1.44,-0.05)** | -0.04(-0.74,0.65) | 0.01(-0.21,0.23) | 0.17(-1.47,1.81) |  |
| Tei index(%) | 0.83(-3.52,5.17) | 0.07(-1.22,1.37) | 0.14(-1.27,1.56) | 0.17(-0.24,0.58) | -2.59(-5.92,0.74) |  |
| E/A | 0.04(-0.15,0.23) | 0.01(-0.06,0.08) | 0.03(-0.03,0.08) | 0.01(-0.02,0.03) | 0.10(-0.10,0.31) |  |
| CO(L/min) | 0.39(-0.53,1.30) | **0.28(0.04,0.52)** | -0.02(-0.21,0.18) | -0.05(-0.13,0.03) | 0.37(-0.62,1.36) |  |
| SV(ml) | 6.49(-2.27,15.25) | **5.14(2.55,7.74)** | 0.40(-1.43,2.24) | -0.38(-1.19,0.43) | 2.33(-8.41,13.07) |  |
| TPVR(dyne*s/cm^5^) | -194.04(-519.88,131.79) | -81.88(-183.59,19.83) | -31.50(-138.41,75.41) | 19.81(-11.68,51.30) | -197.17(-507.70,113.36) |  |
| EDV(ml) | -0.33(-5.56,4.89) | **4.97(3.20,6.74)** | -0.25(-1.55,1.05) | 0.37(-0.21,0.95) | 1.97(-2.48,6.41) |  |
| ESV(ml) | 1.48(-0.84,3.81) | **2.30(1.53,3.06)** | -0.07(-0.75,0.61) | 0.10(-0.15,0.35) | 0.27(-1.88,2.42) |  |
| VTI(cm) | 0.21(-1.91,2.33) | 0.20(-0.50,0.90) | -0.18(-0.82,0.47) | -0.11(-0.32,0.11) | 1.04(-2.15,4.23) |  |
| GLS(%) | 1.29(-1.42,3.99) | **-0.69(-1.31,-0.06)** | -0.20(-0.78,0.38) | -0.08(-0.29,0.13) | -0.87(-4.94,3.20) |  |

The data were presented as β(95%CI) in linear regression models. The missing values were not inputted (Unadjusted: n=943, Adjusted: n=786).

The bold values were P<0.05.

Adjusted for maternal nationality, scholarship, income, HDP, GDM, drink history, passive smoke history, gender of children.

BW: birthweight, GA: gestational age, SBP: systolic blood pressure, DBP: diastolic blood pressure, LV: left ventricle, IVSd: ventricle interventricular septal thickness in diastole, LVIDd: LV internal diameter in diastole, LVPWd: LV posterior wall thickness in diastole, IVSs: ventricle interventricular septal thickness in systole, LVIDs: LV internal diameter in systole, LVPWs: LV posterior wall thickness in systole, RWT: relative wall thickness, LVM: left ventricle mass, LVMI: LV mass index, cIMT: carotid artery intima-media thickness, LVEF: LV ejection fraction, LVFS:LV fractional shorting, CO: cardiac output, SV: stroke volume, TPVR: total peripheral vascular resistance , MAP: mean arterial pressure, EDV: end diastolic volume, ESV: end systolic volume, VTI: velocity time integral, GLS: global longitudinal strain, HDP: hypertensive disorders in pregnancy, GDM: gestational diabetes mellitus.

Table S7. The independent and interaction effect of BW and GA on BP, LV structure and function after multiple imputation.

|  | BW | | | GA | | |  |
| --- | --- | --- | --- | --- | --- | --- | --- |
|  | Crude Model | Model 1 | Model 2 | Crude Model | Model 1 | Model 2 | P for interaction# |
| **Blood pressure** |  |  |  |  |  |  |  |
| SBP (mmHg) | 0.79(-0.24,1.81) | 0.48(-0.54,1.50) | -0.38(-1.36,0.60) | **-0.30(-0.58,-0.02)** | **-0.29(-0.58,-0.01)** | -0.22(-0.49,0.04) | 0.29 |
| DBP (mmHg) | -0.47(-1.29,0.36) | -0.44(-1.28,0.39) | -0.80(-1.63,0.04) | -0.15(-0.38,0.08) | -0.14(-0.38,0.09) | -0.13(-0.36,0.10) | 0.95 |
| MAP (mmHg) | -0.22(-1.00,0.56) | -0.30(-1.09,0.49) | -0.80(-1.58,-0.02) | -0.21(-0.43,0.01) | -0.20(-0.42,0.02) | -0.18(-0.40,0.03) | 0.52 |
| **LV structure** |  |  |  |  |  |  |  |
| IVSd (mm) | 0.04(-0.03,0.12) | 0.03(-0.04,0.11) | 0.03(-0.05,0.11) | -0.01(-0.03,0.01) | -0.01(-0.03,0.01) | -0.01(-0.03,0.01) | 0.42 |
| LVIDd (mm) | **1.04(0.71,1.38)** | **0.87(0.54,1.19)** | **0.63(0.30,0.95)** | **0.11(0.01,0.20)** | **0.10(0.01,0.19)** | **0.10(0.01,0.19)** | **0.01** |
| LVPWd (mm) | **0.13(0.05,0.21)** | **0.11(0.03,0.18)** | **0.08(0.01,0.16)** | -0.01(-0.03,0.01) | -0.01(-0.03,0.01) | -0.01(-0.03,0.01) | 0.32 |
| IVSs (mm) | **0.31(0.18,0.44)** | **0.25(0.12,0.37)** | **0.19(0.06,0.32)** | 0.02(-0.02,0.05) | 0.02(-0.02,0.05) | 0.02(-0.02,0.05) | 0.46 |
| LVIDs (mm) | **0.70(0.44,0.96)** | **0.61(0.35,0.87)** | **0.44(0.18,0.70)** | 0.05(-0.02,0.13) | 0.05(-0.03,0.12) | 0.05(-0.03,0.12) | 0.37 |
| LVPWs (mm) | **0.24(0.12,0.36)** | **0.20(0.08,0.33)** | **0.12(0.00,0.25)** | 0.00(-0.03,0.04) | 0.00(-0.03,0.04) | 0.00(-0.03,0.04) | 0.07 |
| RWT | 0.00(0.00,0.01) | 0.00(0.00,0.01) | 0.00(0.00,0.01) | 0.00(0.00,0.00) | 0.00(0.00,0.00) | 0.00(0.00,0.00) | 0.85 |
| LVM (g) | **2.58(1.78,3.39)** | **2.16(1.36,2.95)** | **1.62(0.83,2.42)** | 0.09(-0.15,0.33) | 0.07(-0.16,0.30) | 0.07(-0.16,0.29) | **0.01** |
| LVMI (g/cm2.7) | **0.66(0.01,1.30)** | 0.44(-0.20,1.09) | 0.07(-0.58,0.72) | 0.09(-0.10,0.28) | 0.08(-0.10,0.27) | 0.08(-0.10,0.27) | 0.43 |
| cIMT (*10-2mm) | 0.28(-0.53,1.08) | 0.27(-0.56,1.09) | 0.22(-0.62,1.06) | 0.11(-0.11,0.32) | 0.09(-0.12,0.31) | 0.09(-0.12,0.31) | 0.19 |
| **LV function** |  |  |  |  |  |  |  |
| LVEF (%) | -0.15(-0.71,0.42) | -0.18(-0.75,0.39) | -0.11(-0.69,0.48) | 0.05(-0.11,0.20) | 0.05(-0.11,0.21) | 0.05(-0.11,0.21) | 0.10 |
| LVFS (%) | -0.03(-0.47,0.42) | -0.07(-0.51,0.38) | -0.02(-0.47,0.44) | 0.04(-0.08,0.17) | 0.05(-0.08,0.17) | 0.05(-0.08,0.17) | 0.08 |
| Tei index (%) | -0.23(-1.06,0.60) | -0.28(-1.12,0.57) | -0.24(-1.10,0.62) | -0.12(-0.36,0.11) | -0.13(-0.36,0.11) | -0.13(-0.37,0.11) | 0.24 |
| E/A | 0.00(-0.04,0.04) | 0.00(-0.04,0.05) | 0.01(-0.03,0.05) | 0.00(-0.01,0.02) | 0.00(-0.01,0.02) | 0.00(-0.01,0.02) | 0.85 |
| CO (L/min) | **0.24(0.08,0.40)** | **0.18(0.02,0.34)** | 0.09(-0.07,0.24) | -0.01(-0.05,0.03) | -0.01(-0.05,0.03) | -0.01(-0.05,0.03) | 0.99 |
| SV (ml) | **3.91(2.21,5.61)** | **0.18(0.02,0.34)** | **2.18(0.51,3.84)** | 0.07(-0.40,0.54) | 0.05(-0.42,0.52) | 0.07(-0.38,0.52) | 0.71 |
| TPVR (dyne*s/cm^5^) | **-77.19(-141.11,-13.26)** | -59.20(-123.00,4.59) | -41.85(-106.22,22.53) | 6.20(-11.09,23.48) | 5.83(-11.35,23.00) | 5.01(-12.05,22.06) | 0.68 |
| EDV (ml) | **3.74(2.56,4.93)** | **3.12(1.96,4.28)** | **2.24(1.10,3.38)** | **0.36(0.01,0.70)** | **0.33(0.00,0.67)** | **0.33(0.01,0.65)** | **0.01** |
| ESV (ml) | **1.37(0.87,1.88)** | **1.19(0.69,1.69)** | **0.85(0.36,1.35)** | 0.09(-0.05,0.23) | 0.08(-0.06,0.22) | 0.08(-0.06,0.22) | 0.36 |
| VTI (cm) | 0.02(-0.43,0.46) | -0.06(-0.52,0.39) | -0.31(-0.76,0.14) | -0.05(-0.17,0.08) | -0.06(-0.18,0.07) | -0.05(-0.18,0.07) | 0.49 |
| GLS (%) | -0.15(-0.56,0.26) | -0.09(-0.50,0.32) | 0.04(-0.38,0.45) | 0.06(-0.05,0.18) | 0.05(-0.07,0.17) | 0.04(-0.07,0.16) | 0.09 |

The data were presented as β(95%CI) in linear regression models. The data inputted missing values with multiple imputation (n=943).

The bold values were P<0.05.

Model 1: adjusted for maternal nationality, scholarship, income, HDP, GDM, drink history, passive smoke history and gender of children.

Model 2: Model 1 + BMI at 4 years old.

#Adjusted for maternal nationality, scholarship, income, HDP, GDM, drink history, passive smoking, gender of children and BMI at 4.

BW: birthweight, GA: gestational age, SBP: systolic blood pressure, DBP: diastolic blood pressure, LV: left ventricle, IVSd: ventricle interventricular septal thickness in diastole, LVIDd: LV internal diameter in diastole, LVPWd: LV posterior wall thickness in diastole, IVSs: ventricle interventricular septal thickness in systole, LVIDs: LV internal diameter in systole, LVPWs: LV posterior wall thickness in systole, RWT: relative wall thickness, LVM: left ventricle mass, LVMI: LV mass index, cIMT: carotid artery intima-media thickness, LVEF: LV ejection fraction, LVFS:LV fractional shorting, CO: cardiac output, SV: stroke volume, TPVR: total peripheral vascular resistance , MAP: mean arterial pressure, EDV: end diastolic volume, ESV: end systolic volume, VTI: velocity time integral, GLS: global longitudinal strain, BMI: body mass index, HDP: hypertensive disorders in pregnancy, GDM: gestational diabetes mellitus.

Table S8. The cardiovascular risk with GA and BW after multiple imputation.

|  | Prehypertension(N=141) | | | Hypertension(N=101) | | | LVH(N=55) | | |
| --- | --- | --- | --- | --- | --- | --- | --- | --- | --- |
|  | Crude | Model 1 | Model 2 | Crude | Model 1 | Model 2 | Crude | Model 1 | Model 2 |
| BW | 0.99(0.89,1.10) | 0.99(0.88,1.10) | 0.99(0.89,1.12) | 0.93(0.83,1.05) | 0.96(0.85,1.08) | 0.97(0.85,1.09) | 1.60(0.84,3.04) | 1.00(1.00,1.00) | 1.59(0.68,3.73) |
| GA | 0.73(0.50,1.13) | 0.71(0.47,1.03) | **0.64(0.42,0.97)** | 1.16(0.70,1.76) | 1.02(0.57,1.84) | 0.80(0.44,1.45) | 1.10(0.91,1.33) | 1.05(0.82,1.33) | 1.05(0.82,1.34) |

Data were presented as OR (95%CI). P for multiplicative and addictive interaction of BW and GA were not significant. The data inputted missing values with multiple imputation (n=943).

The bold values were P<0.05.

Model 1: adjusted for maternal nationality, scholarship, income, HDP, GDM, drink history, passive smoke history and gender of children.

Model 2: Model 1 + BMI at 4 years old.

The 90th and 95th percentile of SBP and DBP for the sex and height were defined according to the Chinese standard (24). Prehypertension was defined as P95th > SBP and/or DBP ≥ P90th. Hypertension was defined as SBP and/or DBP ≥ P95th. LV hypertrophy (LVH) was defined as LVMI ≥ the sex-specific P95th of LVMI.

BW: birthweight, GA: gestational age, LVH: left ventricle hypertrophy, HDP: hypertensive disorders in pregnancy, GDM: gestational diabetes mellitus.
